# Melanoma stem cells drive macrophage reprogramming to a hybrid phenotype, modulating melanoma stemness and compromising NK cell-mediated immunity

**DOI:** 10.3389/fimmu.2026.1698412

**Published:** 2026-06-02

**Authors:** Martina Anselmi, Michela Francesconi, Tiziana Triulzi, Giancarla Bernardo, Valentino Le Noci, Francesca Bianchi, Lorenzo Castagnoli, Francesca Arnaboldi, Nicoletta Gagliano, Serenella M. Pupa, Patrizia Limonta, Lucia Sfondrini, Michele Sommariva

**Affiliations:** 1 Microambiente e Biomarcatori dei Tumori solidi, Dipartimento di Oncologia Sperimentale, Fondazione IRCCS Istituto Nazionale dei Tumori di Milano, Milan, Italy; 2 Dipartimento di Scienze Biomediche per la Salute, Università degli Studi di MIlano, Milan, Italy; 3 Dipartimento di Scienze Farmacologiche e Biomolecolari Rodolfo Paoletti, Università degli Studi di Milano, Milan, Italy

**Keywords:** cancer stem cells, *in vitro* model, macrophages, melanoma, monocyte, NK cells

## Abstract

**Introduction:**

Melanoma stem cells may contribute to tumor progression not only through intrinsic plasticity, but also by shaping the immune microenvironment. However, their interaction with the monocyte-macrophage compartment remains poorly understood.

**Methods:**

Melanosphere cultures derived from A375 and WM115 melanoma cell lines were used as an *in vitro* model enriched for stemness-associated features. THP-1 monocytes were differentiated under a standardized low-dose PMA protocol and exposed to stem cell-conditioned media. Monocyte migration, macrophage transcriptomic and secretory profiling, the effect of macrophage-conditioned medium on NK cytotoxicity and melanoma stem cell phenotype and exploratory clinical relevance in a melanoma patients’ cohort were assessed.

**Results:**

Both melanosphere cell models were enriched for stemness-associated programs. Stem cell-conditioned media promoted THP-1 monocyte migration, which was reduced by CCR2 inhibition. RNA-seq showed that stem cell-conditioned media induced a shared but non-canonical macrophage phenotype enriched for inflammatory, interferon-related, pro-angiogenic, and immune-regulatory programs. Conditioned media from stem cell-educated macrophages impaired NK-cell cytotoxicity through heat-labile soluble mediators and induced cell line-dependent changes in melanoma stemness-associated transcriptional regulators. In an independent melanoma cohort, a shared stem cell-educated macrophage score was associated with inflammatory macrophage programs and with shorter overall survival.

**Conclusions:**

Melanoma stem cells actively shape the monocyte-macrophage compartment by promoting monocyte recruitment and inducing an inflammatory and immunomodulatory macrophage program coupled with downstream effects on NK-cell function and melanoma cell-state regulation. These findings support a bidirectional melanoma stem cell-macrophage axis that warrants validation in more physiological systems.

## 1 Introduction

Cutaneous melanoma is an aggressive cancer characterized by high heterogeneity and phenotypic plasticity. In addition to its complex mutational landscape, melanoma progression is strongly influenced by the ability of tumor cells to switch between different cellular states, a feature that contributes to invasion, metastasis, and resistance to therapy. This dynamic plasticity has increased interest in melanoma cell populations with stemness-associated traits, as they may contribute to tumor progression and adaptation (1–3).

Melanoma stem cells (SCs) are generally considered as a subpopulation of tumor cells characterized by stemness-associated properties (3). Their intratumor abundance is not fixed and increasing evidence suggests that stemness in melanoma is a dynamic and context-dependent cell state that tumor cells may acquire or lose in response to intrinsic and microenvironmental factors (4). Their biological relevance therefore lies less in their absolute abundance than in their ability to sustain tumor growth, adaptive plasticity, dissemination, and treatment resistance. This view helps reconcile earlier hierarchical models with more recent evidence of dynamic cell-state interconversion in melanoma (3, 4).

A growing body of evidence indicates that melanoma SCs do not act only through cell-intrinsic mechanisms but can also actively remodel the tumor microenvironment. Through the release of soluble mediators and other extracellular signals, they may influence surrounding stromal and immune cells and contribute to the establishment of a tumor-supportive niche. In melanoma, this aspect is particularly relevant because phenotype plasticity and tumor microenvironment (TME) interactions are tightly interconnected. However, the specific contribution of melanoma stem-like cells to immune remodeling is still poorly understood (5, 6).

Macrophages represent one major cell component of the melanoma TME and can influence several aspects of disease progression, including tumor growth, invasion, angiogenesis, immune evasion, and response to therapy (7, 8). Because of their marked plasticity, they can respond to local signals in very different ways (9). Historically, macrophages were mainly described within the M1/M2 framework, but this classification is now considered too simplistic to capture their biological complexity in tumors. More recent transcriptomic studies have shown that tumor-associated macrophages (TAMs) comprise multiple functional states, including inflammatory, interferon-responsive, pro-angiogenic, and immune-regulatory programs (10). In melanoma, as in other tumor types, they are therefore better viewed as dynamic and heterogeneous components of the microenvironment rather than as immune cells confined to a simple binary polarization model.

Despite the growing interest in both melanoma SCs and macrophage heterogeneity, the functional interaction between these two cell compartments remains incompletely understood. To address these questions, in the present study we utilized melanosphere cultures generated under our experimental conditions as an *in vitro* model enriched for stemness-associated features and investigated their effects on the monocyte-macrophage compartment using a standardized THP-1-derived macrophage system. By combining transcriptomic, secretory, and functional approaches, we explored how melanoma SCs shape macrophage behavior and how this crosstalk may affect NK-cell cytotoxicity, used here as a functional readout of macrophage-mediated immunomodulatory activity, and melanoma SC transcriptional programs.

## 2 Materials and methods

### 2.1 Cell lines and cell culture

A375 human melanoma cells (CRL-1619™), THP-1 human acute monocytic leukemia cells (TIB-202™), NK-92 human natural killer cell line (CRL-2407™), and K562 human chronic myelogenous leukemia cells (CCL-243™) were purchased from the American Type Culture Collection (ATCC, Manassas, VA, USA). WM115 human melanoma cells were provided by Prof. Patrizia Limonta. A375 cells were cultured in DMEM supplemented with GlutaMAX™, 10% FBS (all from Gibco, Thermo Fisher Scientific, Waltham, MA, USA). WM115 cells were grown in MEM supplemented with GlutaMAX™, 10% FBS (all from Gibco, Thermo Fisher Scientific, Waltham, MA, USA). THP-1 cells were grown in RPMI 1640 supplemented with 4.5 g/L D-Glucose, 2.383 g/L HEPES Buffer, L-Glutamine, 1.5 g/L Sodium Bicarbonate, 110 mg/L Sodium Pyruvate, 10% FBS (all from Gibco, Thermo Fisher Scientific, Waltham, MA, USA). NK-92 cells were grown in alpha-MEM supplemented with 12.5% FBS, 12.5% horse serum, 0.2mM inositol, 0.02mM Folic Acid (all from Gibco, Thermo Fisher Scientific, Waltham, MA, USA) and 200U/ml IL-2 (Miltenyi Biotec, Bergisch Gladbach, Germany). K562 cells were grown in RPMI 1640 supplemented with GlutaMAX™, 10% FBS (all from Gibco, Thermo Fisher Scientific, Waltham, MA, USA). All the cell lines were routinely maintained at 37°C in a 5% CO_2_ atmosphere. The cell lines had been authenticated using short tandem repeat profiling at the Fondazione IRCCS Istituto Nazionale dei Tumori di Milano (Milan, Italy) within the previous 3 years. All experiments were performed with mycoplasma-free cells, as determined with a MycoAlert™ PLUS Mycoplasma Detection Kit (Lonza, Basel, Switzerland).

### 2.2 Generation of melanoma SCs

The generation of melanospheres was performed as previously described (11). Briefly, 5 × 10^5^ melanoma cells were seeded in a 25-cm² flask (Thermo Fisher Scientific, Waltham, MA, USA) in serum-free Euromed-N medium (Euroclone, Pero, Italy), supplemented with 10 ng/mL EGF (Thermo Fisher Scientific, Waltham, MA, USA), 10 ng/mL FGF-2 (Thermo Fisher Scientific, Waltham, MA, USA) and 1% N2 supplement (Thermo Fisher Scientific, Waltham, MA, USA). To maintain SC enrichment, fresh Euromed-N medium was added every 72 h. To prevent necrosis, floating spheres were harvested and mechanically dissociated by pipetting every 15 days. Melanospheres were collected and centrifuged at 200 x g for 5 min, then resuspended in melanosphere-conditioned culture medium (CM) mixed with fresh Euromed-N at a 1:3 ratio and re-plated in new 25-cm² flasks.

### 2.3 Generation of THP-1 derived macrophages and IL-4 polarization

To induce differentiation of THP-1 monocytes into macrophage-like cells, THP-1 cells were seeded at different cellular densities depending on the specific experiment. Specifically, 3.5×10^5^ cells were seeded in 12-well plates (Thermo Fisher Scientific, Waltham, MA, USA), while 5×10^5^ cells were plated in 6-well plates (Thermo Fisher Scientific, Waltham, MA, USA), in the presence of 20, 80 or 160 nM Phorbol 12-myristate 13-acetate (PMA) (Sigma-Aldrich, Merck, Darmstadt, Germany) for 72 hours. Macrophages generated under these conditions were considered unpolarized macrophages (M0). For polarization experiments, M0 macrophages were incubated for 24 h in complete medium containing 20 ng/mL IL-4 (PeproTech, Thermo Fisher Scientific, Waltham, MA, USA). Control macrophages were incubated with fresh medium only. Under all experimental protocols, PMA was maintained in the culture medium.

### 2.4 Preparation of CM from melanoma SCs

A375 and WM115 SCs, obtained as described above, were seeded in 25-cm^2^ flasks (Thermo Fisher Scientific, Waltham, MA, USA) at a density of 8×10^5^ cells and incubated in fresh Euromed-N (Euroclone, Pero, Italy) for 24 h without growth factors. The supernatant was then collected, centrifuged at 4400 rpm for 3 min and stored at -80°C until use. THP-1-derived macrophages were incubated with melanoma SC-CM containing 20 nM PMA for 24 or 48 h, washed with PBS, and used for subsequent analysis. M0 cells that received fresh Euromed-N (Euroclone, Pero, Italy) without growth factors and 20 nM PMA served as controls.

### 2.5 RNA sequencing

Total RNA was extracted using Direct-zol RNA Microprep kit (Zymo Research, Irvine, CA, USA) according to the manufacturer’s instructions. Sequencing libraries were prepared from 100 ng of total RNA using the QuantSeq 3’ mRNA-Seq V2 Library Prep Kit FWD (Lexogen GmbH, Vienna, Austria), as per the manufacturer’s instructions. After a quality check on the 4150 TapeStation System (Agilent Technologies, Inc, Santa Clara, CA, USA), libraries were equimolarly pooled and sequenced on Illumina NovaSeq X Plus System (Illumina, Inc., San Diego, CA, USA).

### 2.6 Bioinformatic analysis

FASTQ files were processed using the QuantSeq 3′ mRNA-seq pipeline implemented on theBlueBee Genomics Platform (Lexogen). Data were aligned through Lexogen’s data analysis platform to the GRCh38/hg38 assembly human reference genome. For melanoma cell lines grown under 3D and 2D conditions and for SC-educated macrophages, two separate raw count matrices were generated and analyzed independently in R using the DESeq2 package (v1.46.0) from Bioconductor. Low expressed genes were filtered out, and principal component analysis (PCA) was performed to explore the overall variability among samples. Differential gene expression analysis was performed and genes with an adjusted p-value (padj) < 0.05 were considered significantly differentially expressed (DEG). Gene set enrichment analysis (GSEA) was performed using the fgsea package (v1.36.2) against up-regulated DEGs on Reactome pathways, with enrichment assessed using 10,000 permutations (nperm = 10,000). Single sample GSEA (ssGSEA) was performed using the GSVA package (v2.2.1) against three collections of gene sets: (i) Reactome pathways, (ii) curated melanoma stemness signature, (iii) literature-derived macrophage/TAM signatures from Ma et al. (10), and (iv) curated macrophage-state signatures derived from an independent single-cell RNA-seq dataset (GSE115978) (12) (**Supplementary Table 1**). To derive the curated macrophage-state signatures, raw count data from the GSE115978 single-cell RNA-seq dataset were re-analyzed using the Scanpy Python library. Macrophage cells were isolated, yielding 420 cells. Genes expressed in fewer than 3 cells and cells expressing fewer than 200 genes were removed, retaining 17,896 genes. Unsupervised clustering was performed using the Leiden algorithm across multiple resolutions; resolution 0.20 was selected as the final clustering, yielding four macrophage clusters. Cluster marker genes were identified using the Wilcoxon rank-sum test on log-normalized expression values (top 50 genes per cluster). One cluster displaying a clear plasmacytoid dendritic cell-like transcriptional profile was excluded from further analyses. Candidate signature genes were selected by retaining only genes with padj < 0.001 and log2 fold change > 1. The resulting candidate gene lists were then subjected to manual biological curation. Genes considered overly generic, poorly informative, associated with immediate-early or stress-response programs, broadly pan-myeloid but non-discriminative, potentially contaminating, or excessively related to housekeeping or lysosomal functions were excluded. This procedure yielded three curated macrophage programs, designated FCN1+IL1B+ TAMs, C1QC+APOE+ TAMs, and FOLR2+SEPP1+ TAMs. Curated macrophage signatures were named using representative marker genes with established relevance to macrophage subsets described in cancer (13–18). The online gene-list analysis tool Metascape (https://metascape.org/gp/index.html#/main/step1) was used to functionally annotate the curated macrophage-state signatures derived from the single-cell RNA-seq dataset, to identify the biological functions and processes associated with each gene set. Analyses were performed using the default parameters. Relationships among the signatures were evaluated by correlation analysis of ssGSEA scores across all samples using Spearman’s rank correlation coefficient. For each pairwise comparison, p values were computed and adjusted for multiple testing using the Benjamini-Hochberg procedure. To assess similarity in gene composition among the signatures, overlapping coefficient was defined as the ratio between the number of genes shared by two signatures and the size of the smaller signature. Transcription factor (TF) activity was estimated using the VIPER algorithm (Virtual Inference of Protein-activity by Enriched Regulon analysis), through the viper R package (v1.42.0). Regulon networks were derived from the DoRothEA resource (dorothea_hs), retaining only TF-target interactions with high-confidence evidence levels. VIPER was applied to the log2-normalized expression matrix, using the “scale” method with nes = TRUE and the minimal regulon size filter set to 10. To identify transcriptomic signatures discriminating SC-educated macrophages from unpolarized M0 macrophages, penalized logistic regression with a LASSO penalty (alpha = 1) was performed using the glmnet R package (v4.1.10) on the set of DEGs shared between the two SC-CM versus M0 comparisons (padj<0.05). The resulting signature was applied to GSE98394 cohort (19). For each primary melanoma patient, a signature score was calculated. Patients were then stratified using the first quartile of the score distribution, with the lowest quartile defining tumors with a more M0-like profile and the upper three quartiles defining tumors with a more SC-educated macrophage-like profile. Kaplan-Meier survival curves were estimated using the survival R package (v3.8.3).

### 2.7 Preparation of CM from macrophages

THP-1 cells were seeded in 12-well plates (Thermo Fisher Scientific, Waltham, MA, USA) at a density of 3 × 10^5^ cells/well and differentiated as described above. For collection of supernatants from IL-4-treated macrophages, M0 macrophages were treated with 20 ng/mL IL-4 for 48 h in the presence of 20 nM PMA, washed with PBS, and then incubated for an additional 24 h in serum-free RPMI 1640. For collection of CM from melanoma SC-educated macrophages, M0 macrophages were exposed to melanoma SC-CM for 24 h in the presence of 20 nM PMA, washed with PBS, and then incubated for an additional 24 h in fresh Euromed-N without growth factors. In each case, control M0 macrophages were processed under the same medium conditions used for the corresponding experimental group. Supernatants were then collected, centrifuged at 4400 rpm for 3 min to remove cell debris, and stored at −80°C until use. 

### 2.8 Flow cytometry analysis

THP-1 cells were seeded in 6 well-plates (5×10^5^ cells/well) (Thermo Fisher Scientific, Waltham, MA, USA) and differentiated as described above. After exposure to different PMA concentrations or to 20 ng/mL IL-4 (Peprotech, Thermo Fisher Scientific, Waltham, MA, USA), THP-1 monocytes were collected and THP-1-derived macrophages were detached from the plate using Versene Solution (Gibco, Thermo Fisher Scientific, Waltham, MA, USA). Collected cells were washed with PBS and then incubated with LIVE/DEAD™ Fixable Near IR (780) (Invitrogen, Thermo Fisher Scientific, Waltham, MA, USA) following manufacturer’s instructions. Cells were resuspended in PBS containing 1% FBS and stained for 30 min at 4°C with the following directly conjugated primary antibodies: PerCP/Cyanine5.5 anti-human CD33 (clone: P67.6), FITC anti-human CD14 Antibody (clone: HCD14), Alexa Fluor^®^ 700 anti-human CD11b (clone: ICRF44), APC anti-human HLA-DR (clone: LN3), PE anti-human CD80 (clone: 2D10), PE/Cyanine7 anti-human CD86 (clone: BU63) (all from BioLegend, San Diego, CA, USA). BD OptiBuild™ BV605 Mouse Anti-Human CD206 (clone: 15.2) and BD Horizon™ BV421 Mouse Anti-Human CD163 (clone: MAC2-158) antibodies (BD Biosciences, Franklin Lakes, NJ, USA) were diluted in Brilliant Stain Buffer (Invitrogen, Thermo Fisher Scientific, Waltham, MA, USA) following manufacturer’s protocol. The cells were then washed twice with PBS, fixed with 10% neutral buffered formalin solution (Thermo Fisher Scientific, Waltham, MA, USA) for 15 min at room temperature in the dark, washed and then resuspended in PBS. All samples were acquired with a BD LSRFortessa instrument and analyzed using the FlowJo software (version 10.10.0) (BD Biosciences Franklin Lakes, NJ, USA).

### 2.9 Real-time PCR (RT-qPCR)

THP-1 monocytes were seeded in 6 well-plates (Thermo Fisher Scientific, Waltham, MA, USA) at a density of 5 × 10^5^ cells/well and differentiated as described above. After incubation with different PMA concentrations, IL-4 or melanoma SC-CM in the presence of 20 nM PMA for 24 h, cells were washed twice with PBS. Total RNA was extracted using Direct-zol RNA Microprep kit (Zymo Research, Irvine, CA, USA) according to the manufacturer’s instructions. A High-Capacity RNA-to-cDNA Kit (Applied Biosystems, Thermo Fisher Scientific, Waltham, MA, USA) was used for mRNA reverse transcription. Real-time PCR analysis was carried out using TaqMan^®^ Fast Universal PCR Master Mix (Applied Biosystems, Thermo Fisher Scientific, Waltham, MA, USA) on a StepOne Real-Time PCR System (Applied Biosystems, Thermo Fisher Scientific, Waltham, MA, USA) using the following TaqMan^®^ gene expression assays (Applied Biosystems, Thermo Fisher Scientific, Waltham, MA, USA): SPI1 (Hs02786711_m1), *MAFB* (Hs00534343_s1), *IRF4* (Hs00180031_m1), STAT6 (Hs00598625_m1), *MRC1* (Hs00267207_m1), CD274 (Hs00204257_m1), CD163 (Hs00174705_m1), KLF4 (Hs00358836_m1), SOX10 (Hs00366918_m1), POU5F1 (Hs04260367_gH), SOX9 (Hs00165814_m1), *SOX2* (Hs04234836_s1), HPRT1 (Hs02800695_m1). The expression of each gene was normalized to HPRT1 expression level. PCR data were analyzed using the 2^−ΔCt^ method as previously described (20).

### 2.10 Multiplex ELISA assay

Multiplex protein quantification was performed using a bead-based immunoassay based on Luminex xMAP technology (Bio-Rad, Hercules, CA, USA), as previously described (21, 22), and ProcartaPlex™ Human Inflammation Panel, 20plex (Thermo Fisher Scientific, Waltham, MA, USA) according to the manufacturer’s protocol. Briefly, samples were incubated with the 20-plex beads with analyte-specific capture antibodies. After washing, biotinylated detection antibody mix was added, followed by streptavidin-phycoerythrin (SA-PE) for signal development. Each analyte was identified by a specific bead region, allowing multiplex detection within a single well and enabling simultaneous bead classification and quantification of PE fluorescence intensity. Standard curves were generated using manufacturer-provided standards, covering a broad dynamic range (pg/mL) depending on the analyte. Data acquisition was performed using a dual-laser detection system. Concentrations of samples were calculated by interpolation from a five-parameter logistic (5-PL) standard curve. All samples were analyzed in duplicate, and assay performance was validated using internal controls and manufacturer-provided specifications. Data were processed using Bio-Plex Manager software and expressed as pg/mL. Bioplex data were curated to account for out-of-range (OOR) measurements. Analytes with ≥ 25% OOR values across replicates were excluded from downstream analyses. For the remaining analytes, OOR values were imputed as the upper limit of quantification (ULOQ) or as half of the lower limit of quantification (LLOQ/2), as appropriate. PCA was performed on log-transformed concentrations (log10[x+1]) with centering and unit-variance scaling across analytes using R (version 4.5.3) and RStudio (2026.01.1 Build 403) (Posit PBC, Boston, MA, USA). Comparisons between A375 and WM115 melanosphere CM were performed on the curated analyte matrix using two-sided Mann-Whitney U tests for each analyte. p-values were adjusted using the Benjamini-Hochberg procedure. Comparison among A375 and WM115 SC-educated macrophage and M0 macrophage CM was performed using Kruskal–Wallis tests with Benjamini–Hochberg correction applied across analytes to control the false discovery rate (FDR). Post-hoc pairwise comparisons were then applied using Dunn’s test with Holm adjustment across the three pairwise contrasts.

### 2.11 ELISA assay

Macrophage supernatants were analyzed using ELISA kits for IL-6 (DY206-05), IL-10 (DY217B-05), TNF-α (DY210-05) (R&D Systems, Minneapolis, MN, USA) according to the manufacturer’s instructions. The absorbance was measured using an iMark Microplate Absorbance Reader (Bio-Rad, Hercules, CA, USA).

### 2.12 Chemotaxis assay

Monocyte recruitment was evaluated using transwell filters with an 8 µm pore size polycarbonate membrane (Corning Incorporated, Corning, NY, USA). The lower chamber was filled with A375 or WM115 SC-CM or with fresh Euromed-N supplemented with 0.5% BSA (Merck KGaA, Darmstadt, Germany) as control medium. THP-1 cells were harvested, counted, and resuspended in RPMI 1640 supplemented with 0.5% BSA. A total of 2 × 10^5^ THP-1 cells were seeded into the upper chamber and incubated at 37°C in a humidified atmosphere containing 5% CO_2_ for 4 h. To evaluate the involvement of CCR2 in THP-1 chemotaxis toward melanoma SC-CM, THP-1 cells were pre-treated with 2 µM of CCR2 antagonist RS504393 (R&D Systems, Minneapolis, MN, USA) for 60 min at 37°C prior to seeding. RS504393 was also added to the lower chamber to maintain constant inhibitor exposure throughout the assay. At the end of the experiment, migrated cells were collected from the lower chamber and quantified by manual counting using a hemocytometer. Results were expressed as the number of migrated cells per well.

### 2.13 Effect of SC-educated macrophage CM on melanoma SCs

5×10^5^ A375 and WM115 cells per well were seeded into low-attachment 6-well plates (Corning Incorporated, Corning, NY, USA) and cultured for 7 days in CM derived from THP-1-derived macrophages previously exposed to melanoma SC-CM. Macrophage CM was supplemented with stem cell growth factors as described above. After 7 days, spheres were collected and Real-Time PCR analysis for melanoma SC-associated genes was carried out.

### 2.14 NK-cell cytotoxicity assay

NK-92 cells were incubated overnight with CM from macrophages previously exposed to A375 or WM115 melanoma SC-CM, at a density sufficient to yield the required number of effector cells for the cytotoxicity assay. Macrophage CM was used at 50% (v/v) by mixing 1:1 with NK-92 assay medium (RPMI 1640 without phenol red with 1% FBS), and IL-2 was adjusted to a final concentration of 200 U/mL in all conditions. Supernatants from M0 macrophages served as controls. The following day, NK-92 cells were collected, washed once with PBS, counted, and resuspended at the appropriate concentrations in assay medium for the cytotoxicity assay. K562 target cells were harvested, washed with PBS, and resuspended at 1×10^6^ cells/mL in assay medium. Calcein-AM (Invitrogen, Thermo Fisher Scientific, Waltham, MA, USA) was added to a final concentration of 5 µg/mL, and cells were incubated for 30 min at 37°C in the dark with gentle mixing every 10–15 min. Cells were washed three times with assay medium by centrifugation at 130 × g for 10 min at room temperature. After the final wash, labeled cells were resuspended at 1×10^6^ cells/mL in assay medium and allowed to rest for 30 min at 37°C in the dark to reduce spontaneous dye release. Cells were washed once more, resuspended in assay medium, counted, and adjusted to the working concentration for plating. Cytotoxicity assays were performed in 96-well round-bottom plates (Corning Incorporated, Corning, NY, USA). Calcein-labeled K562 target cells were co-cultured with NK-92 effector cells at effector-to-target (E:T) ratios of 100:1, 50:1, and 25:1, with six technical replicates per E:T ratio and condition, in a final volume of 200 µL per well. Plates were incubated for 4 h at 37°C, 5% CO_2_ , protected from light. At the end of the incubation, plates were centrifuged, and supernatants were transferred to black, optical-bottom 96-well plates (Nunc™, Thermo Fisher Scientific, Waltham, MA, USA). Spontaneous release controls consisted of target cells incubated without effectors, while maximum release controls consisted of target cells lysed with 2% Triton X-100 (Merck KGaA, Darmstadt, Germany). Fluorescence was measured using a GloMax^®^ Discover Detection System (Promega, Madison, WI, USA) with calcein-compatible settings (fluorescence excitation: 495 nm; fluorescence emission: 516 nm). The percentage of specific lysis was calculated as: 100 × [(F_sample − F_spontaneous)/(F_maximum − F_spontaneous)], where F_sample is the fluorescence measured in co-cultures, F_spontaneous is the fluorescence of target-only wells, and F_maximum is the fluorescence following complete target lysis. For heat treatment of CM, aliquots (0.5 mL) of the different macrophage-CM were transferred into microcentrifuge tubes and incubated at 80°C for 15 min in an Eppendorf Thermomixer Comfort (Eppendorf, Hamburg, Germany). Samples were then immediately cooled on ice for 10 min and centrifuged at 16,000 x g for 20 min to pellet heat-denatured material. The supernatant was carefully collected without disturbing the pellet and used for subsequent experiments.

### 2.15 Statistical analysis

Statistical analyses were performed using GraphPad Prism (GraphPad Software, San Diego, CA, USA), R (version 4.5.3) and RStudio (2026.01.1 Build 403) (Posit PBC, Boston, MA, USA). When applicable, data distribution was assessed for normality before selecting the appropriate statistical test. Comparisons between two groups were performed using Welch’s t-test, whereas nonparametric data were analyzed using the Mann–Whitney U test. Comparisons among three or more groups were performed using one-way ANOVA followed by Tukey’s multiple-comparison test, or by Kruskal–Wallis test followed by Dunn’s multiple-comparison test for nonparametric data. When two independent variables were analyzed, two-way ANOVA followed by the appropriate *post hoc* multiple-comparison test was used. When multiple endpoints were tested within the same experiment, p values were adjusted for multiple comparisons using Holm or Benjamini–Hochberg correction. Data are presented as mean ± SEM and differences were considered statistically significant at p < 0.05.

## 3 Results

### 3.1 Transcriptomic profiling of melanoma SCs reveals stemness enrichment and cell line-specific features

**Figure 1:**
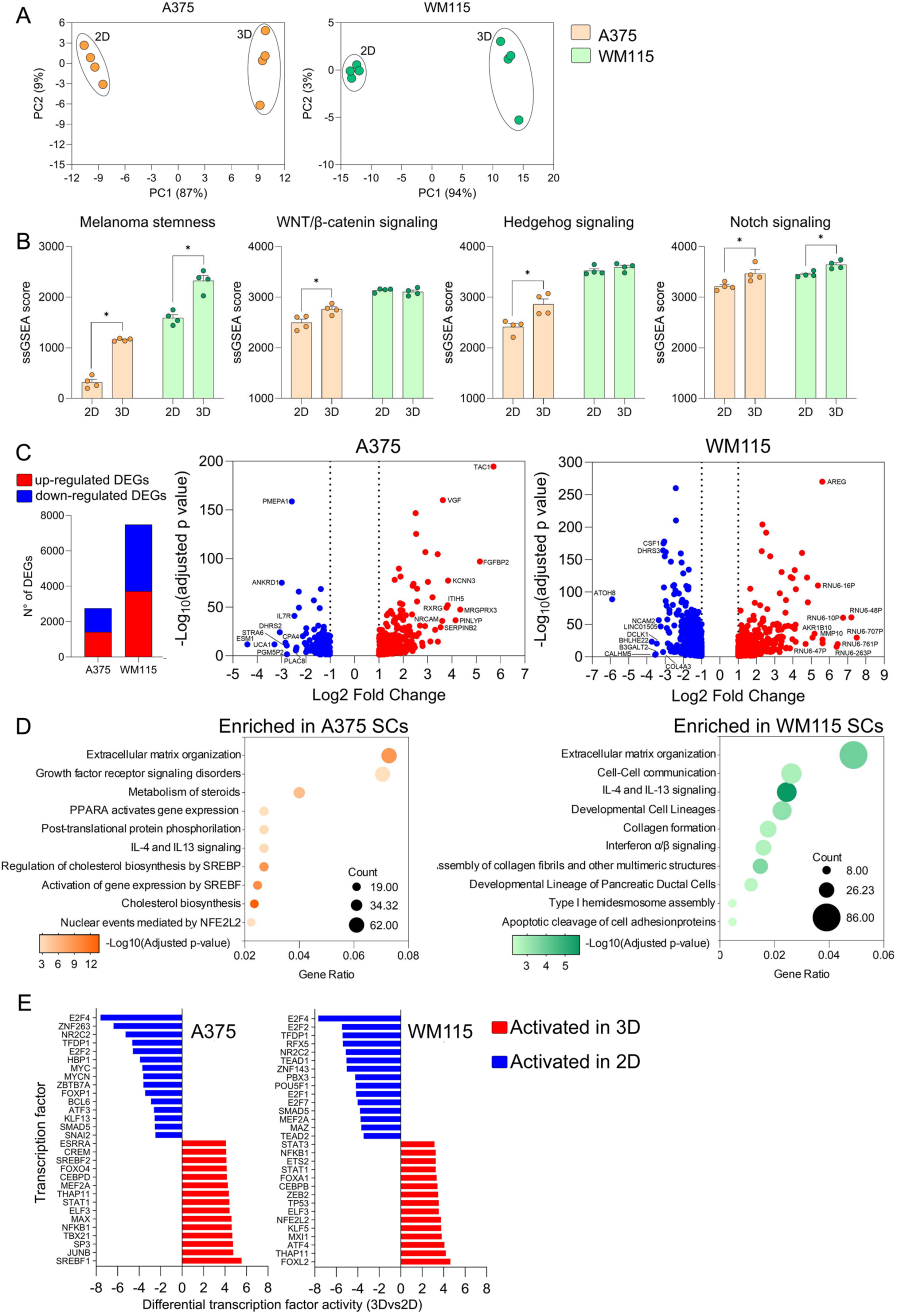
Transcriptomic profiling of melanoma SCs identifies stemness-associated and cell line-specific molecular programs. **(A)** PCA of RNA-seq data from A375 and WM115 cells cultured under adherent conditions (2D) or as melanospheres (3D). Each dot represents one sample. **(B)** ssGSEA enrichment scores for the Melanoma stemness, WNT/β-catenin signaling, Hedgehog signaling, and Notch signaling gene sets in A375 and WM115 cells cultured in 2D or 3D conditions. Data are presented as the mean ± SEM. Each dot represents one sample. *p<0.05 by Welch’s t-test, followed by Benjamini-Hochberg correction for multiple testing across signatures. **(C)** Left, number of up-regulated and down-regulated DEGs identified in melanospheres compared to matched adherent cultures in A375 and WM115 cells (padj < 0.05). Right, filtered volcano plots showing DEGs (padj < 0.05, |log2FC| > 1, and baseMean > 50) in A375 and WM115 melanospheres relative to 2D cultures. The 10 most upregulated and 10 most downregulated genes are labelled. **(D)** Bubble plots of the top 10 most statistically significant (padj < 0.05) Reactome pathways enriched among genes up-regulated in A375 and WM115 melanospheres relative to matched 2D cultures determined by GSEA analysis. The X-axis represents the Gene Ratio and Y-axis indicates enriched pathway terms. Bubble area represents the gene count and bubble color indicates the adjusted p value. **(E)** VIPER analysis showing the top 15 inferred transcription factor activity in melanospheres relative to matched 2D cultures in A375 and WM115 cells. Red bars indicate transcription factors inferred to be activated in 3D cultures, whereas blue bars indicate transcription factors inferred to be activated in 2D cultures.

**Figure 2:**
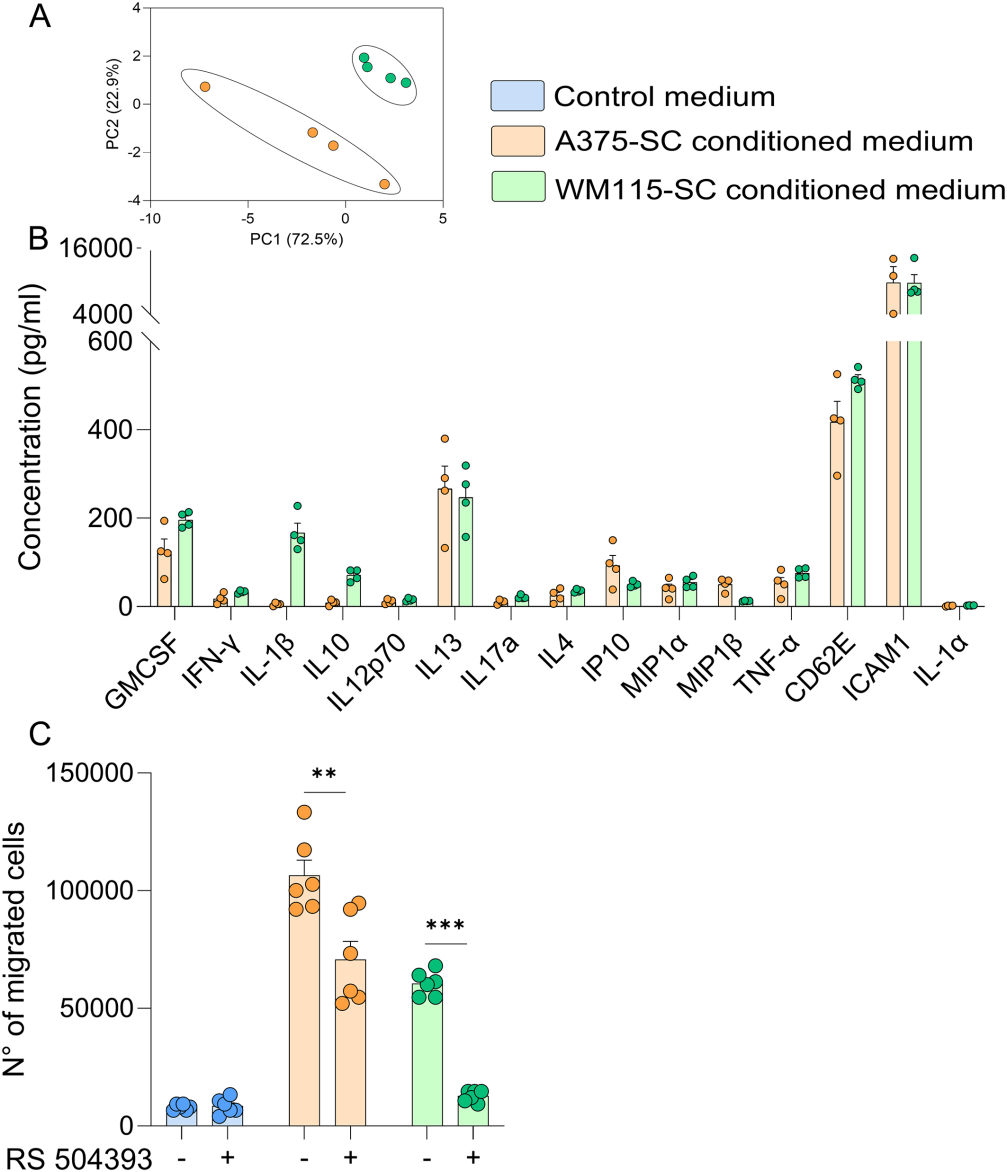
Distinct secretory profiles and monocyte chemoattractant capacity of A375 and WM115 SCs. **(A)** PCA of multiplex ELISA assay data obtained from CM of A375 and WM115 melanospheres. Each dot represents one sample. **(B)** Multiplex ELISA assay quantification of soluble factors in CM from A375 and WM115 SCs. Data are presented as the mean ± SEM. Each dot represents one sample. **(C)** Transwell migration assay of THP-1 cells exposed to control medium, A375 SC-CM, or WM115 SC-CM, in the absence or presence of the CCR2 antagonist RS504393. Data are presented as mean ± SEM. Each dot represents one sample. Statistical analysis was performed by two-way ANOVA followed by Bonferroni-adjusted *post hoc* comparisons. Asterisks indicate comparisons between inhibitor-treated and untreated conditions within each medium. **p < 0.01, ***p < 0.001.

To provide a comprehensive molecular characterization of A375 and WM115 SCs, RNA-seq profiling of melanospheres and the parental cell lines was performed. PCA analysis showed a clear segregation between 2D and 3D cultures in both A375 and WM115 cells, indicating that 3D growth is associated with a distinct transcriptional profile compared with adherent conditions (**Figure 1A**). To determine whether the *in vitro*-generated SCs displayed stemnessfeatures, ssGSEA analysis was performed using a melanoma stemness gene set containing genes previously reported to be characteristic of melanoma stem-like cells (**Supplementary Table 1**) (3, 23). This analysis revealed that SCs are significantly more enriched in melanoma stemness signature than their corresponding 2D cell counterparts. Moreover, SCs derived from the A375 cell line showed significant enrichment of WNT/β-catenin, Hedgehog, and Notch signaling pathways, all reported to be implicated in melanoma SC biology (4, 6), whereas in WM115 cells only the Notch pathway was significantly increased under 3D cell culture conditions (**Figure 1B**). We then compared SCs with matched 2D cultures within each cell line. In A375 cells, we identified 1,409 upregulated and 1,345 downregulated DEGs (padj < 0.05) in SCs compared with adherent cultures, while in the WM115 cell line 3,714 upregulated and 3,767 downregulated DEGs (padj < 0.05) (**Figure 1C**). The complete list of DEGs is provided in **Supplementary Table 2**. GSEA analysis of genes upregulated in A375 melanospheres included enrichment of pathways related to cholesterol and steroid metabolism. These metabolic pathways were accompanied by enrichment of NFE2L2-mediated nuclear events, PPARA-dependent transcription, IL-4/IL-13 signaling, and extracellular matrix organization. In WM115 melanospheres, the significantly enriched gene pathways were primarily related to extracellular matrix organization and adhesion. In addition, WM115 showed enrichment of interferon alpha/beta signaling and IL-4/IL-13 signaling (**Figure 1D**). The complete list of statistically significant enriched pathways is provided in **Supplementary Table 2**. VIPER analysis, performed to infer the upstream regulatory circuits associated with these transcriptional changes, revealed a marked rewiring of transcription factor activity in melanospheres compared with matched 2D cell cultures. In A375 cells, melanosphere growth was associated with increased activity of regulators such as SREBF1, JUNB, NFKB1, ELF3, STAT1, and MEF2A paralleled by a reduced activity of E2F4, TFDP1, E2F2, MYC, and MYCN. In WM115 cells, melanospheres similarly displayed strong repression of E2F-centered programs, including E2F4, E2F2, TFDP1, and E2F1, but were characterized by increased activity of different transcription factors, i.e. FOXL2, ATF4, NFE2L2, TP53, ZEB2, and CEBPB (**Figure 1E**). To determine whether these transcriptional differences were also reflected at the level of secreted factors, we next profiled the CM of A375 and WM115 melanospheres using a multiplex cytokine panel. PCA indicated that the secretory profiles of the two melanosphere models were distinguishable, consistent with the presence of line-specific differences in soluble factor production (**Figure 2A**). Although no analyte remained significantly different between the two experimental groups after correction for multiple testing, WM115 melanosphere-CM showed numerically higher levels of several inflammatory and immunomodulatory mediators, most notably IL-1β and IL10, consistent with a more prominent inflammatory secretory profile. In contrast, A375 SC-CM displayed a more selective pattern, most notably characterized by higher MIP-1β levels and, to a lesser extent, increased IP-10 (**Figure 2B**). Given that MCP-1/CCL2 is a potent monocyte chemoattractant (24) and was abundantly present in CM from both SC models, exceeding the upper limit of quantification in all samples (data not shown), we next investigated whether melanosphere-derived CM could functionally promote monocyte migration. Using a transwell assay, we found that CM from both A375 and WM115 melanospheres significantly increased THP-1 migration compared with control medium (both p<0.001 by Bonferroni-adjusted *post hoc* pairwise comparisons following two-way ANOVA). Notably, A375 SC-CM induced a stronger migratory response than WM115 SC-CM (p<0.001 by Bonferroni-adjusted *post hoc* pairwise comparisons following two-way ANOVA). To assess whether this chemoattractant activity was mediated, at least in part, through the CCL2-CCR2 axis, THP-1 cells were pre-incubated with the CCR2 antagonist RS504393 before the migration assay. CCR2 inhibition significantly reduced THP-1 migration induced by both melanosphere-CMs, while having no effect under control conditions (**Figure 2C**).

### 3.2 Optimization of THP-1 differentiation into macrophage-like cells and evaluation of IL4 responsiveness under continuous PMA exposure

**Figure 3:**
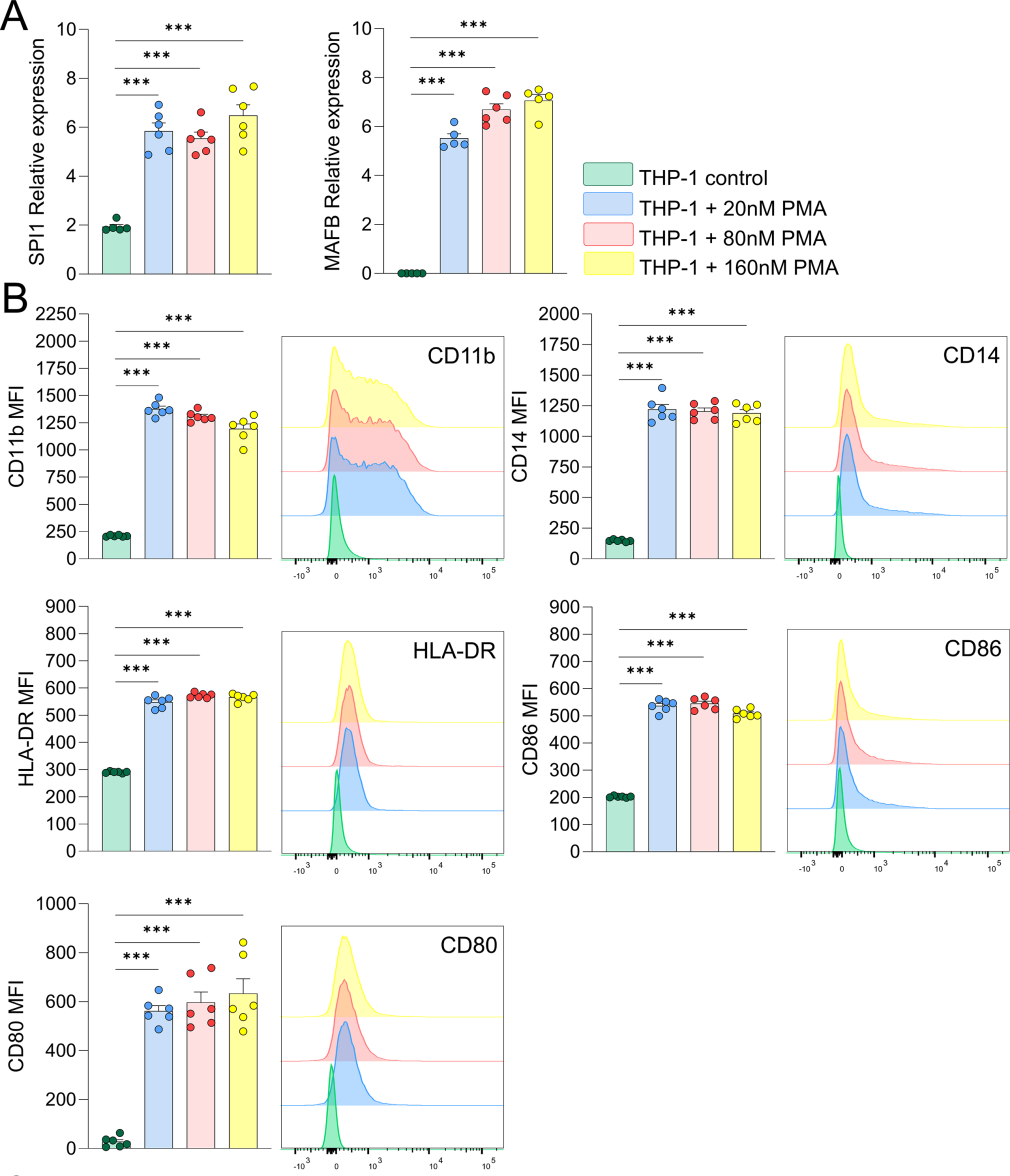
Optimization of PMA-driven THP-1 differentiation toward a macrophage-like phenotype. **(A)** RT-qPCR analysis of SPI1 and MAFB expression in untreated THP-1 cells and in THP-1 cells treated with 20, 80, or 160 nM PMA for 72 h. Bar plots represent 2^−ΔCt^ RT-qPCR values. **(B)** Flow-cytometric analysis of CD11b, CD14, HLA-DR, CD80, and CD86 in untreated THP-1 cells and in THP-1 cells treated with 20, 80, or 160 nM PMA for 72 h. Bar plots represent mean fluorescence intensity (MFI) of each marker. MFI values refer to the live CD33+ population. Representative histogram overlays are shown alongside each marker. Data are presented as mean ± SEM. Each dot represents one sample. The results are representative of one of at least three independent experiments showing similar results. Statistical analysis was performed by one-way ANOVA followed by Tukey’s *post hoc* test, after Holm correction across endpoints. Asterisks indicate comparisons with untreated control. Only comparisons versus untreated control are displayed in the graph, whereas significant differences among PMA concentrations are reported in the text. ***p<0.001.

To identify the most appropriate conditions for THP-1 differentiation toward macrophage-like cells, we compared three PMA concentrations (20, 80, and 160 nM for 72 h). Cell adherence to the culture plate surface and acquisition of an elongated morphology were the initial indicators of the monocyte-to-macrophage differentiation process, as previously described (25). All tested PMA concentrations induced cell adherence to the culture plate, with no detectable differences in the morphology of the developing macrophages observed either throughout the incubation period or after 72 hours (data not shown). RT-qPCR analysis showed that both SPI1 and MAFB, genes relevant for macrophage differentiation (26, 27), were significantly upregulated in all PMA-treated conditions relative to untreated THP-1 cells, whereas no significant differences were observed among the three PMA concentrations (**Figure 3A**). Flow-cytometric analysis demonstrated a significant increase in CD11b, CD14, HLA-DR, CD80, and CD86, markers of macrophage differentiation and activation (28), in cells exposed to 20, 80, or 160 nM PMA compared with untreated controls. Notably, increasing PMA concentration did not confer a uniform phenotypic advantage. For instance, CD11b expression was significantly lower at 160 nM than at 20 nM (padj = 0.0011) and 80 nM (padj = 0.0457), while CD86 was also reduced at 160 nM compared with 20 nM (padj = 0.0481) and 80 nM (padj = 0.0080). By contrast, HLA-DR expression was modestly but significantly higher at 80 nM than at 20 nM (padj = 0.0247), whereas no significant differences among PMA concentrations were detected for CD14 or CD80 (**Figure 3B**). Overall, these data identify 20 nM PMA as a sufficient and appropriate condition to generate THP-1-derived macrophage-like cells that were considered as M0 macrophages.

**Figure 4:**
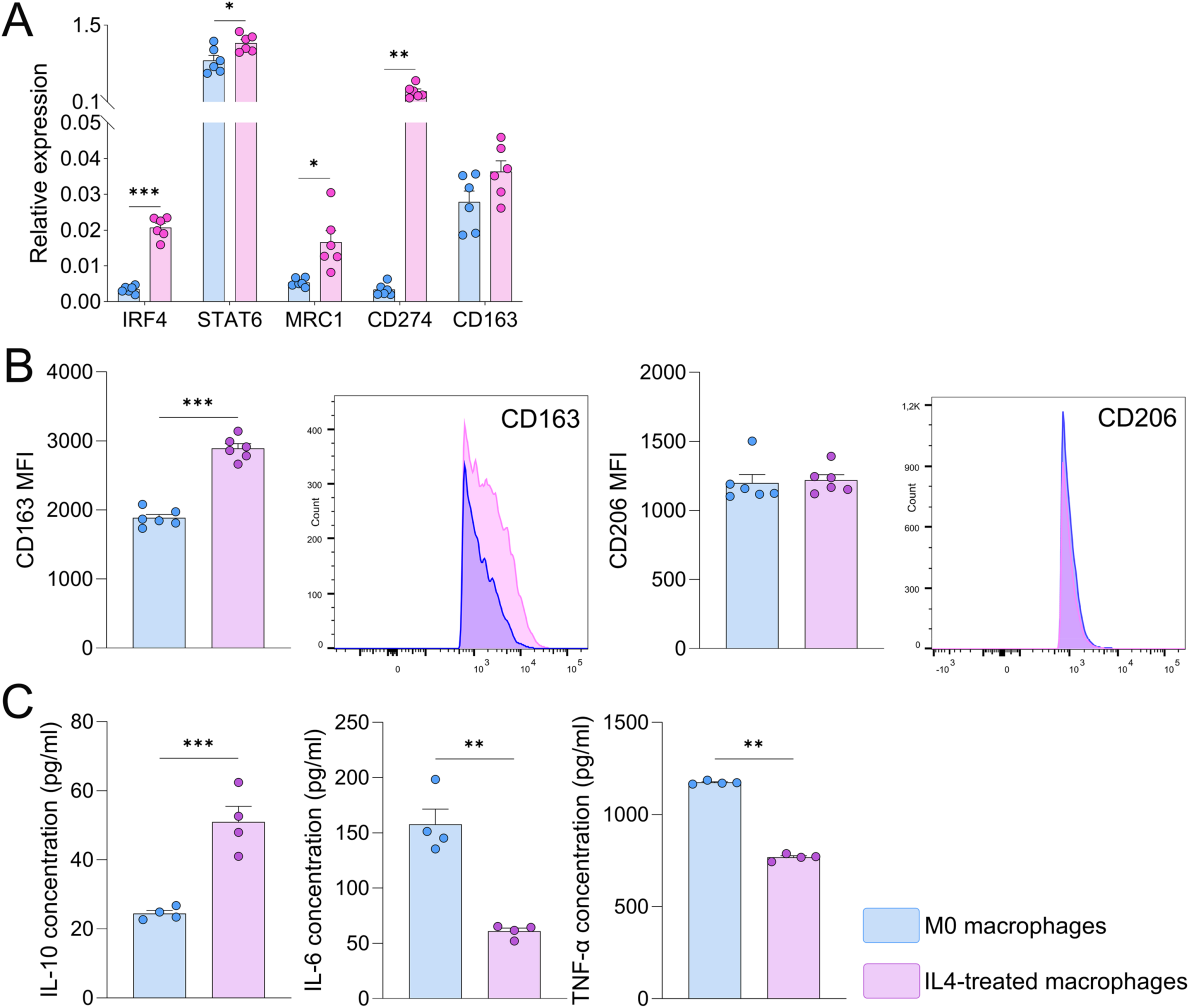
Assessment of IL4-responsive activation in THP-1-derived macrophages under continuous PMA exposure. **(A)** RT-qPCR analysis of IRF4, STAT6, MRC1, CD274, and CD163 in THP-1-derived macrophages maintained under continuous PMA exposure and cultured either in basal conditions (M0) or after IL4 treatment. Bar plots represent 2^−ΔCt^ RT-qPCR values. **(B)** Flow-cytometric analysis of CD163 and CD206 in M0 and IL4-treated THP-1-derived macrophages maintained under continuous PMA exposure. Bar plots represent MFI of each marker. MFI values refer to the live population. Representative histogram overlays are shown alongside each marker. **(C)** ELISA quantification of IL-10, IL-6, and TNF-α in supernatants from M0 and IL4-treated THP-1-derived macrophages maintained under continuous PMA exposure. Data are presented as mean ± SEM. Each dot represents one sample. The results are representative of one of at least three independent experiments showing similar results. Statistical analysis was performed by Welch’s t-test, followed by Holm correction for multiple testing across endpoints. *p<0.05, **p<0.01, ***p<0.001.

Since it has been reported that one of the major limitations of using the THP-1 cell line is that once PMA is removed from culture, dedifferentiation of the cells can occur (29), we maintained PMA in the culture medium throughout the duration of the experiments. However, since PMA can induce a pro-inflammatory phenotype in macrophages (25), we initially verified whether the continuous presence of PMA interferes with the acquisition of an immunoregulatory/tissue-repair phenotype. Despite continuous PMA exposure, RT-qPCR analysis indicated that IL4-treated cells retained the ability to upregulate genes associated with alternative macrophage activation (30), including IRF4, STAT6, MRC1 and CD274 whereas CD163 expression was not altered (**Figure 4A**). Flow-cytometric analysis showed increased CD163 protein levels following IL4 treatment, while CD206 remained unchanged (**Figure 4B**). ELISA assays further demonstrated that IL4 increased IL10 release while reducing the secretion of the pro-inflammatory cytokines TNF-α and IL6 (**Figure 4C**). Overall, these findings indicate that 20 nM PMA represents a suitable condition for generating THP-1-derived macrophage-like cells and that continuous low-dose PMA exposure does not abolish their subsequent phenotypic plasticity.

### 3.3 Melanoma SC-CM induces a distinct inflammatory and immunomodulatory state in THP-1-derived macrophages

**Figure 5:**
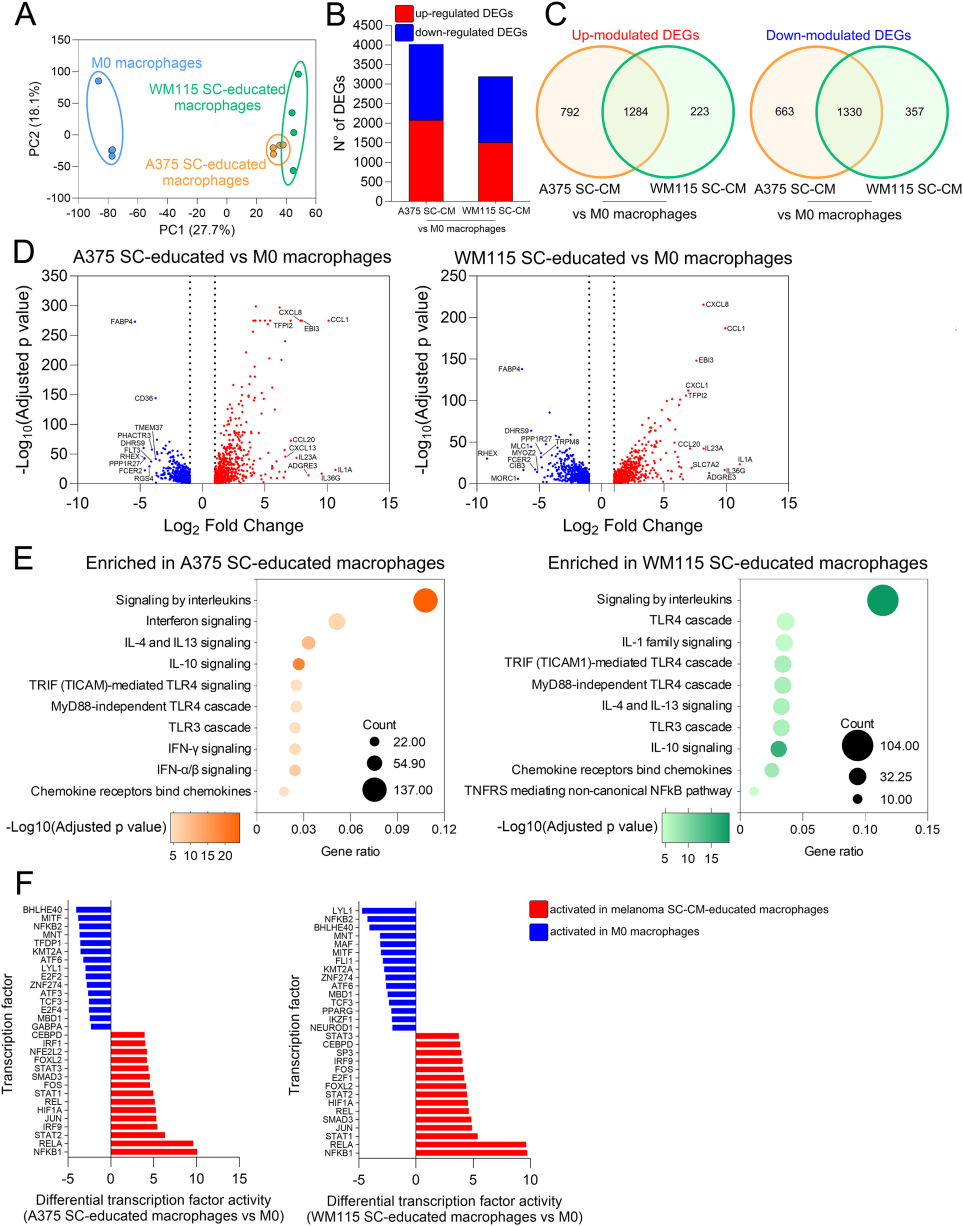
Transcriptomic characterization of THP-1-derived macrophages exposed to melanoma SC-CM.**(A)** PCA of RNA-seq data from M0 macrophages and macrophages exposed to CM from A375 or WM115 melanospheres. **(B)** Number of upregulated and downregulated DEGs (padj<0.05) in A375 SC-CM-educated and WM115 SC-CM-educated macrophages compared with M0 macrophages. **(C)** Venn diagrams showing the overlap of upregulated and downregulated DEGs between A375 SC-CM-educated and WM115 SC-CM-educated macrophages, each compared with M0 macrophages. **(D)** Filtered volcano plots showing DEGs (padj < 0.05, |log2FC| > 1, and baseMean > 50) in A375 SC-CM-educated versus M0 macrophages and WM115 SC-CM-educated versus M0 macrophages. The 10 most upregulated and 10 most downregulated genes are labelled. **(E)** Bubble plots of the top 10 most statistically significant (padj < 0.05) Reactome pathways enriched among genes upregulated in A375 SC-CM-educated macrophages and WM115 SC-CM-educated macrophages determined by GSEA analysis. The X-axis represents the Gene Ratio and Y-axis indicates enriched pathway terms. Bubble area represents the gene count, and bubble color indicates the adjusted p value. **(F)** VIPER analysis showing the top 15 inferred transcription factor activity in A375 SC-educated and WM115 SC-educated macrophages compared to M0 macrophages. Red bars indicate transcription factors inferred to be activated in melanoma SC-educated macrophages, whereas blue bars indicate transcription factors inferred to be activated in M0 macrophages.

**Figure 6:**
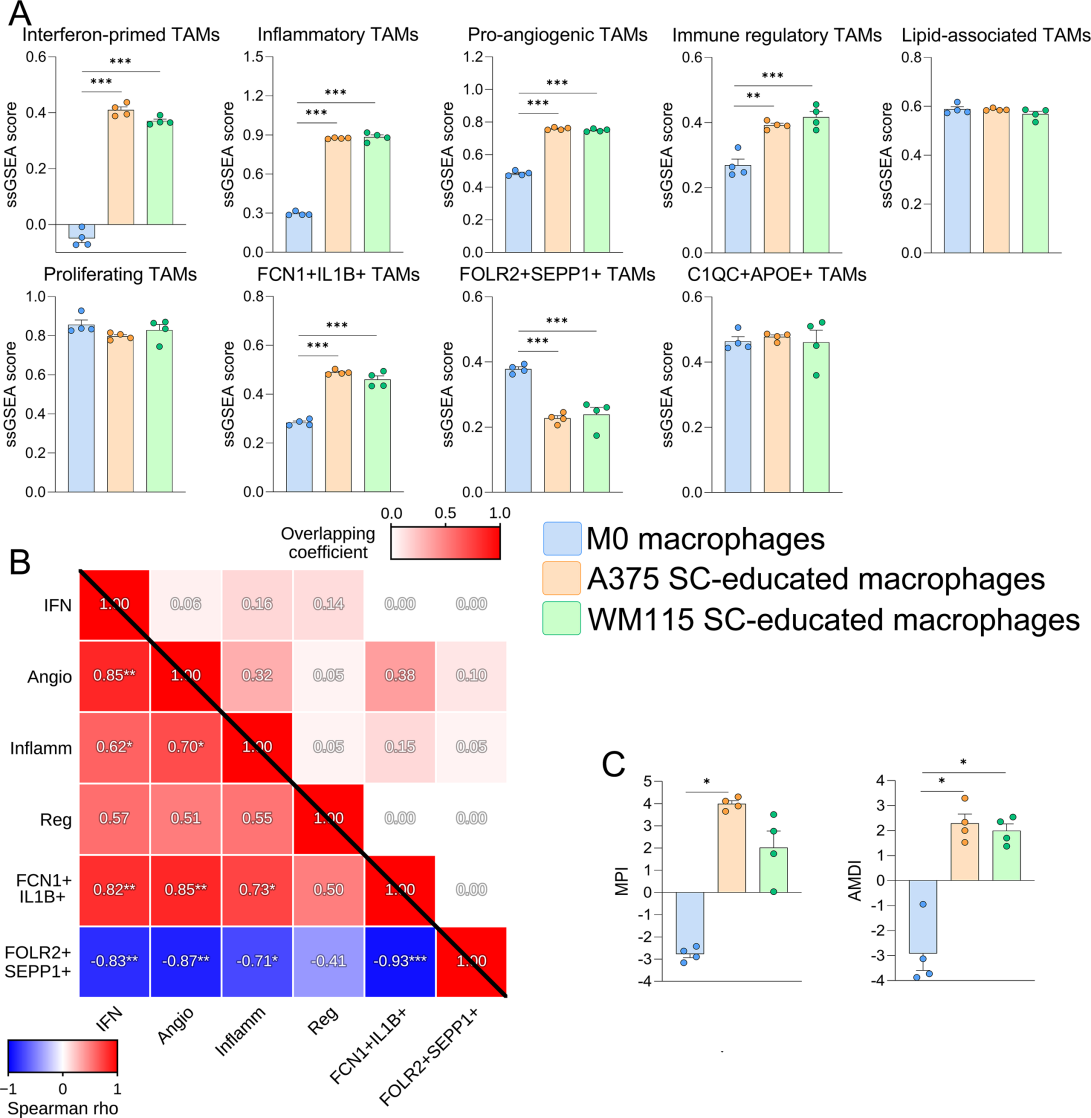
ssGSEA-based inference of macrophage phenotypes induced by melanoma SC-CM. **(A)** Bar plots showing normalized ssGSEA scores for literature-derived TAM signatures and curated macrophage-state signatures in M0 macrophages and in macrophages exposed to A375 SC-CM or WM115 SC-CM. Curated signatures were derived from GSE115978. Data are presented as the mean ± SEM. Each dot represents one sample. **p<0.01, ***p<0.001 by one-way ANOVA with Benjamini-Hochberg correction across signatures followed by Tukey’s multiple-comparison test. **(B)** Integrated matrix showing pairwise relationships among the signatures significantly modulated *in vitro*. The lower triangle reports Spearman correlation coefficients calculated from ssGSEA scores across samples, with asterisks indicating Benjamini-Hochberg-adjusted significance levels (*p<0.05, **p<0.01, ***p<0.001). The upper triangle reports overlap coefficients based on gene-set composition. Abbreviations: IFN, interferon-primed TAMs; Inflamm, inflammatory cytokine-enriched TAMs; Angio, pro-angiogenic TAMs; Reg, immune regulatory TAMs. **(C)** Bar plots representing MPI (macrophage polarization index) and AMDI (activation-induced macrophage differentiation index) values from MacSpectrum analysis of M0 macrophages and macrophages exposed to CM from A375 or WM115 melanospheres. Data are presented as the mean ± SEM. Each dot represents one sample. *p<0.05, **p<0.01 by Kruskal-Wallis test with Holm correction across the two endpoints, followed by Dunn’s *post hoc* test with Holm correction.

**Figure 7:**
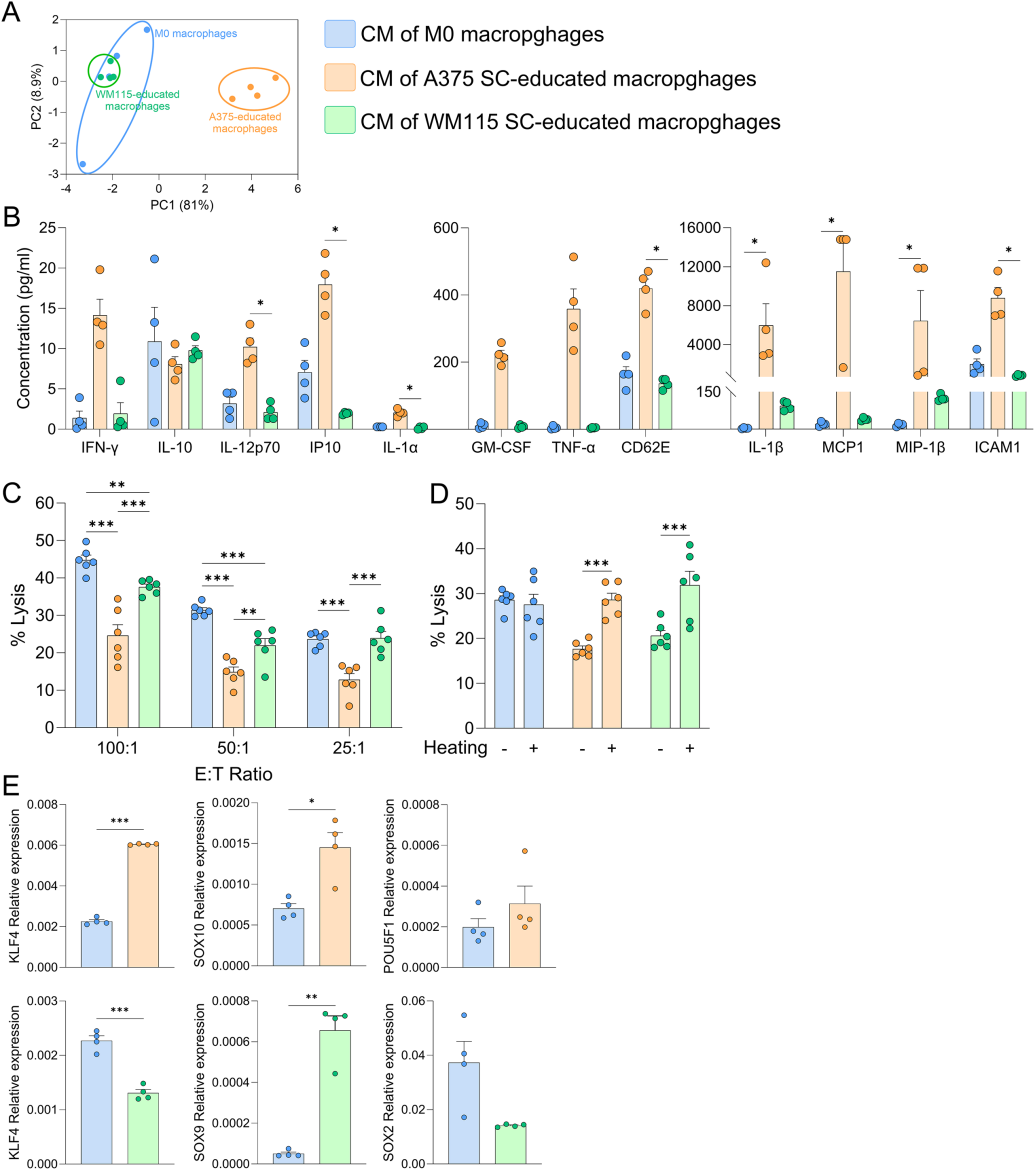
Functional characterization of THP-1-derived macrophages exposed to melanoma SC-CM. **(A)** PCA performed on multiplex ELISA assay of soluble mediator profiles from M0 macrophages and macrophages exposed to CM from A375 or WM115 melanospheres. Each dot represents one sample. **(B)** Multiplex ELISA assay quantification of soluble mediators secreted by M0 macrophages and by macrophages previously exposed to CM from A375 or WM115 melanospheres. Data are presented as the mean ± SEM. Each dot represents one sample. *p<0.05 by Kruskal-Wallis test with Benjamini-Hochberg correction across analytes, followed by Dunn’s *post hoc* test with Holm correction; pairwise p values were additionally Benjamini-Hochberg-corrected across analytes within each contrast. **(C)** Percentage of lysis of NK-92 cells against K562 target cells after pre-exposure to CM from M0 macrophages or from macrophages educated with A375 or WM115 SC-CM, assessed by Calcein-AM release cytotoxicity assay at the indicated E:T ratios. Data are presented as the mean ± SEM. Each dot represents one sample. The results are representative of one of at least three independent experiments showing similar results. **p<0.01, ***p<0.001 by Tukey-adjusted comparisons among experimental groups within each E:T ratio following two-way ANOVA. **(D)** Percentage of lysis of NK-92 cells against K562 target cells after pre-exposure to untreated or heat-treated CM from M0 macrophages or from macrophages educated with A375 or WM115 melanoma SC-CM assessed by Calcein-AM release cytotoxicity assay. Data are presented as the mean ± SEM. Each dot represents one sample. The results are representative of one of at least three independent experiments showing similar results. ***p<0.001 by Tukey-adjusted comparisons between treatment conditions within each experimental group following two-way ANOVA. **(E)** RT-qPCR analysis of KLF4, SOX10, POU5F1, SOX9, SOX2 in A375 and WM115 SC-enriched cultures after exposure to CM from M0 macrophages or from macrophages previously educated with melanoma SC-CM. Bar plots show mean ± SEM of 2^−ΔCt^ RT-qPCR values. The results are representative of one of at least three independent experiments showing similar results. *p<0.05, **p<0.01, ***p<0.001 by Welch’s t-test, followed by Holm correction for multiple testing across genes within each cell line.

Given the central role of soluble factors in orchestrating the interplay between SCs and the surrounding immune microenvironment (3), we exposed M0 macrophages to A375 and WM115 SC-CM to explore the ability of melanoma SCs to shape the macrophage phenotype by RNA-seq analysis. PCA showed a clear segregation of control M0 macrophages from SC-CM-exposed macrophages, indicating that both melanoma secretomes profoundly altered the macrophage transcriptome. At the same time, A375 SC-CM- and WM115 SC-CM-treated macrophages formed distinct but related clusters, consistent with the presence of a common response program accompanied by condition-specific features (**Figure 5A**). Differential gene expression analysis revealed a broader transcriptional rewiring in macrophages exposed to A375 SC-CM than in those exposed to WM115 SC-CM. Specifically, A375 SC-CM induced 4069 DEGs (2076 up-modulated and 1993 down-modulated genes at padj < 0.05), whereas WM115-SC CM modulated 3194 genes (1507 up-modulated and 1687 down-modulated genes at padj < 0.05) (**Figure 5B**). Despite the differences, the two conditions shared 2614 DEGs (1284 up-modulated and 1330 down-modulated genes at padj < 0.05), all showing a concordant direction of change (**Figure 5C**). The volcano plot in **Figure 5D** shows the most up- and down-regulated genes and the complete list of DEGs is provided in **Supplementary Table 3**. Pathway enrichment analysis of genes significantly upregulated in THP-1-derived macrophages exposed to melanosphere-CM revealed both shared and cell line-specific responses. In A375 SC-CM-treated macrophages, the most evident cell line-specific feature was the enrichment of interferon-related pathways, whereas WM115 SC-CM-treated macrophages were more characterized by IL1/NF-kB-related signaling. At the same time, several pathways were enriched in both models, including interleukin signaling, chemokine signaling, TNF signaling, Toll-like receptor cascades, and IL-4/IL-13 and IL-10 signaling, supporting the presence of a shared macrophage activation program induced by melanoma SC-CM (**Figure 5E**). The complete list of significantly enriched pathways is provided in **Supplementary Table 3**. VIPER analysis further supported the existence of a shared regulatory program in THP-1-derived macrophages exposed to melanoma SC-CM. Despite some model-specific differences, both conditions converged on a common core of activated transcription factors centered on NF-kB-, AP-1- and STAT-related networks, including regulators such as NFKB1, RELA, REL, JUN, FOS, STAT1, STAT2, STAT3 (31, 32) (**Figure 5F**). Overall, this pattern is consistent with activation of a broader inflammatory and innate immune transcriptional program in macrophages exposed to melanoma SC-derived cues. To characterize the phenotype acquired by SC-educated macrophages, we performed ssGSEA using literature-derived macrophage/TAM signatures (10) together with curated macrophage-state signatures derived from an independent single-cell RNA-seq dataset (GSE115978) (12) (**Supplementary Table 2**). Functional annotation of the curated gene sets by Metascape supported their biological coherence, indicating that FCN1+IL1B+ TAMs were associated with inflammatory and innate effector programs, C1QC+APOE+ TAMs with immunoregulatory and endocytic/antigen-handling functions, and FOLR2+SEPP1+ TAMs with a more resident/homeostatic program (**Supplementary Table 4**). ssGSEA showed that macrophages exposed to either A375 SC-CM or WM115 SC-CM were significantly enriched for interferon-primed TAMs, inflammatory cytokine-enriched TAMs, pro-angiogenic TAMs, and immune regulatory TAMs relative to M0 macrophages, whereas no consistent enrichment was observed for lipid-associated TAMs or proliferating TAMs. In addition, both A375 SC-CM- and WM115 SC-CM-treated macrophages showed enrichment of the FCN1+IL1B+ TAM program together with depletion of the FOLR2+SEPP1+ TAM program, whereas the C1QC+APOE+ TAM program was not significantly enriched (**Figure 6A**). Notably, the overall ssGSEA profile was highly similar between A375 SC-CM- and WM115 SC-CM-treated macrophages, indicating that the two melanoma secretomes converge on a shared macrophage state. Correlation analysis restricted to the signatures significantly modulated *in vitro* further revealed interferon-primed TAMs, inflammatory cytokine-enriched TAMs, pro-angiogenic TAMs, and FCN1+IL1B+ TAMs were positively correlated with one another. By contrast, FOLR2+SEPP1+ TAMs were inversely associated with all the other signatures. To determine whether these coordinated behaviors reflect underlying similarity in gene content, we next quantified pairwise overlap among the same six significant signatures. The strongest overlap was observed between pro-angiogenic TAMs and both FCN1+IL1B+ TAMs and inflammatory cytokine-enriched TAMs, whereas all other pairwise comparisons showed weaker overlap (**Figure 6B**). In addition, MacSpectrum analysis, which maps macrophages along dimensions of inflammatory polarization (MPI) and activation-associated maturation (AMDI) (33), further supported the phenotypic effects of melanoma SC-CM. MPI, a measure of inflammatory polarization, was significantly increased in A375 SC-educated macrophages relative to M0 controls. AMDI, which reflects activation-associated macrophage maturation, was significantly elevated in both A375- and WM115-SC-educated macrophages compared with M0 controls (**Figure 6C**). Collectively, these analyses indicate that melanoma SC-CM drives macrophages toward a shared inflammatory state composed of partially convergent, but non-redundant, TAM-associated transcriptional programs. To determine whether the transcriptional reprogramming induced by melanoma SC-CM was accompanied by functional changes in macrophage secretory activity, we profiled soluble mediators released by M0 macrophages and by macrophages previously exposed to A375 SC-CM or WM115 SC-CM via a multiplex ELISA assay. PCA showed a clear separation of A375 SC-educated macrophages from both M0 and WM115 SC-educated macrophages, whereas the latter remained considerably closer to the M0 condition (**Figure 7A**). MCP-1, MIP-1β, and IL-1β showed the most prominent induction and reached the highest levels in A375 SC-educated macrophages, whereas WM115 SC-educated macrophages displayed only a modest increase. Several additional mediators followed the same overall pattern, with IP-10, ICAM-1, IL-1α, CD62E, and IL-12p70 remaining significantly higher in A375 SC-educated macrophages after multiple-testing correction (**Figure 7B**). These data suggest that melanoma SC-CM can promote an inflammatory remodeling of the macrophage secretome, consistent with the transcriptomic reprogramming observed in SC-educated macrophages, and indicate that A375 SC-CM may exert a stronger effect than WM115 SC-CM in promoting this inflammatory phenotype. Since macrophages are known to shape the antitumor immune response also through the release of soluble factors (7), we next assessed the immunomodulatory activity of the CM of melanoma SC-educated macrophages using NK-cell cytotoxicity as a functional readout. To this end, NK-92 cells were pre-exposed to CM collected from M0 macrophages or from macrophages previously exposed to A375 or WM115 SC-CM, and their cytotoxicity against K562 target cells was assessed by a calcein-AM release assay at increasing E:T ratios. Across all E:T ratios tested, CM from A375 SC-educated macrophages reduced NK-92 cytotoxicity compared with the CM from M0 macrophages, whereas the effect of CM from WM115 SC-educated macrophages was weaker and was evident at E:T 50:1 and 100:1, but not at 25:1 (**Figure 7C**). Given that NK-cell activity can be profoundly influenced by soluble protein mediators, including cytokines and chemokines (34), we next repeated the cytotoxicity assay using CM untreated or heat-inactivated to reduce the biological activity of thermolabile protein factors. Heat treatment had no appreciable effect on CM from M0 macrophages but reduced the inhibitory activity of CM derived from both A375 and WM115 SC-educated macrophages (**Figure 7D**).

We next investigated whether melanoma SC-polarized macrophages could influence the phenotype of melanoma SCs. Therefore, THP-1-derived macrophages were incubated with CM from melanoma SCs as previously described. After 24 h, the CM was replaced with fresh culture medium and allowed to be conditioned by macrophages for an additional 24 h. This macrophage CM was then supplemented with SC-supporting factors and subsequently used in a melanosphere formation assay (**Supplementary Figure 1**). **Supplementary Figure 1** shows representative micrographs of SCs obtained from the two cell lines incubated with CM from M0 or SC- educated macrophages. Morphological analysis of the resulting melanospheres from both cell lines revealed no differences in the sphere formation rate or diameter (data not shown).

We then performed a targeted expression analysis of selected candidate genes identified among the genes upregulated in each melanoma SC model and reported to be characteristic of melanoma SCs (3, 35). In A375 SCs, exposure to CM derived from A375 SC-educated macrophages was associated with significantly increased KLF4 and SOX10 expression compared with CM from M0 macrophages, whereas POU5F1 remained unchanged. In contrast, in WM115 SCs, CM from WM115 SC-educated macrophages induced a significant reduction in KLF4 together with increased SOX9 expression, while SOX2 did not reach statistical significance after multiple-testing correction. These findings indicate that melanoma SC-reprogrammed macrophages can feed back on SC-enriched melanoma populations and modulate stemness-associated transcriptional programs, although the direction and nature of this effect differ between the A375 and WM115 models (**Figure 7E**).

**Figure 8:**
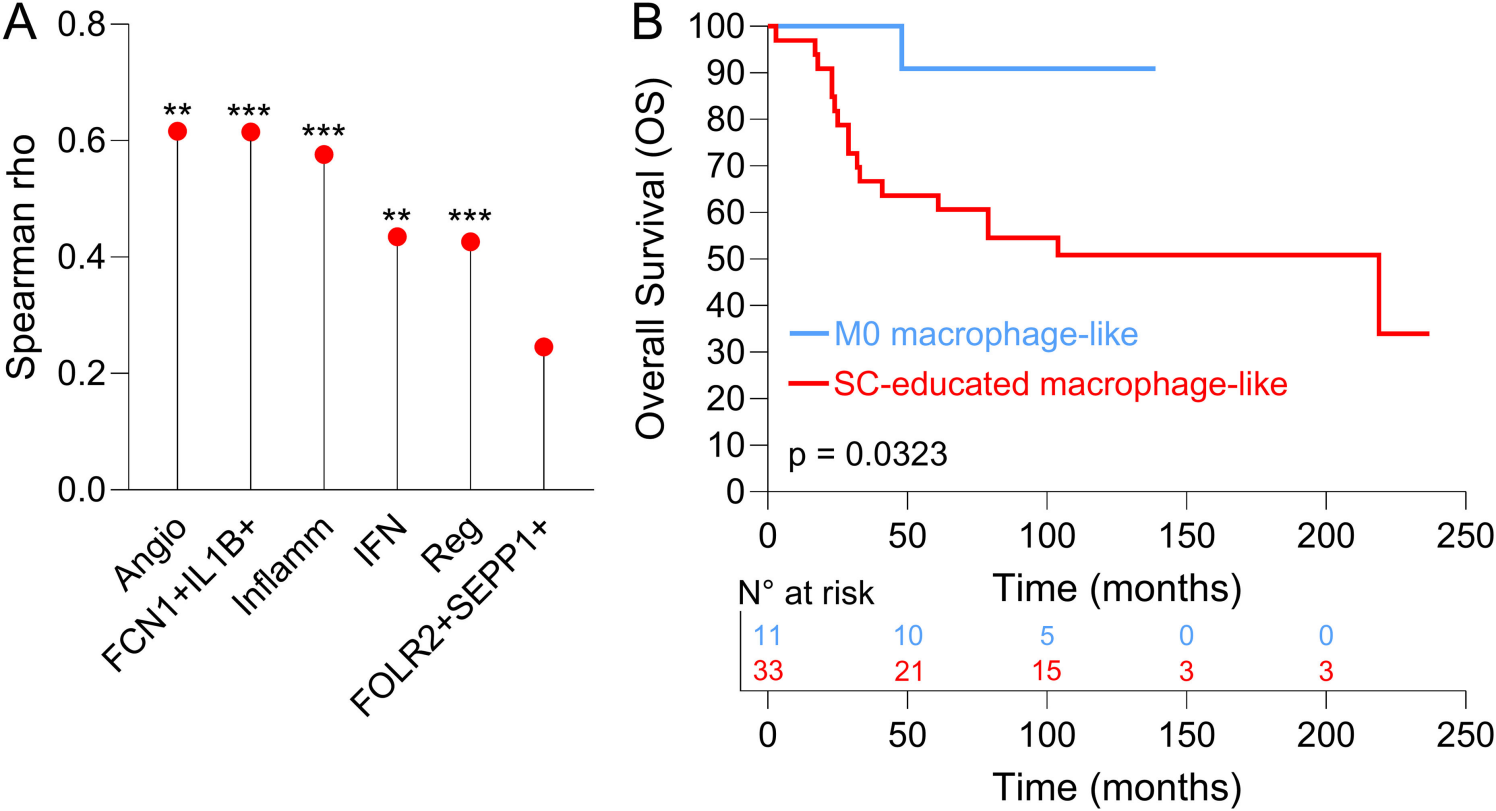
Association of macrophage LASSO score with macrophage signatures and patient survival in primary melanoma. **(A)** Lollipop plot showing correlation between the macrophage LASSO score and ssGSEA scores for the macrophage signatures significantly modulated *in vitro* in the GSE98394 primary melanoma cohort. Correlations were assessed by Spearman’s rank correlation test, with p values adjusted using the Benjamini-Hochberg method (**p<0.01, ***p<0.001). Abbreviations: Angio, pro-angiogenic TAMs; Inflamm, inflammatory cytokine-enriched TAMs; IFN, interferon-primed TAMs; Reg, immune regulatory TAMs. **(B)** Kaplan-Meier overall survival analysis in the GSE98394 primary melanoma cohort stratified according to the LASSO score. Patients in the lowest quartile were classified as M0 macrophage-like, whereas patients in the upper three quartiles were classified as SC-educated macrophage-like. Survival differences were assessed by the log-rank test. The number of patients at risk is shown below the plot.

To explore the potential translational relevance of the macrophage programs identified *in vitro*, we generated a LASSO score reflecting the phenotype of macrophages exposed to melanoma SC-CM. The model was restricted to the transcriptional changes shared between A375 and WM115 SC-educated macrophages compared to M0 macrophages, with the aim of capturing a common SC-educated macrophage program rather than line-specific effects. This LASSO score was then applied to a cohort of primary melanoma samples from GSE98394 (19). First, to determine whether this score reflected the macrophage states identified *in vitro*, we performed ssGSEA in the tumor dataset using only the macrophage signatures that had been significantly modulated in the *in vitro* analyses. Correlation analysis showed that the LASSO score was positively associated with the same inflammatory macrophage programs that emerged *in vitro*. The strongest relationships were observed with pro-angiogenic TAMs, FCN1+IL1B+ TAMs, and inflammatory cytokine-enriched TAMs, whereas more moderate but still significant positive associations were found with interferon-primed TAMs and immune regulatory TAMs. FOLR2+SEPP1+ TAMs were not significantly associated with the score (**Figure 8A**). Overall, these results indicate that the LASSO score captures a macrophage program recapitulating *in vivo* the same transcriptional axis identified in macrophages educated *in vitro* by melanoma SC-CM. In an exploratory analysis, we next asked whether this LASSO score might be associated with patients’ survival. Patients were stratified according to the first quartile of the score, such that the lowest quartile identified tumors with a profile more like M0 macrophages, whereas the upper three quartiles corresponded to tumors with a profile more like SC-educated macrophages. Patients classified as SC-educated macrophage-like showed shorter survival than those classified as M0 macrophage-like (**Figure 8B**). Taken together, these findings suggest that the macrophage program induced *in vitro* by melanoma SC-CM may also be reflected *in vivo*.

## 4 Discussion

Among immune cell populations, macrophages are particularly relevant in melanoma because they are abundant, highly plastic, and deeply integrated into the biology of tumor progression, dissemination, and therapy response (7, 8). However, translating their peculiar characteristics into experimentally robust human *in vitro* systems remains challenging. Primary monocyte-derived macrophages from healthy donors or patients with melanoma would represent the most physiologically relevant model but the inter-donor variability can complicate interpretation and requires large cohorts to obtain reproducible results. Although THP-1-derived macrophages cannot fully recapitulate the biological heterogeneity and donor-dependent variability of primary human monocyte-derived macrophages, this cellular model remains an experimentally suitable model of the human monocyte/macrophage lineage (36–38). Because THP-1 differentiation critically depends on PMA exposure, and existing protocols vary considerably across studies (36–38), we first sought to establish a standardized experimental setting for macrophage generation before investigating their education by melanoma-derived cues.

We first compared three PMA concentrations, including two within the range commonly used in literature and a lower dose. Our data showed that even the lowest concentration tested was sufficient to promote THP-1 differentiation toward macrophage-like cells, as indicated by cell adherence, acquisition of an elongated morphology, and upregulation of canonical differentiation markers. Importantly, increasing PMA concentration did not provide a consistent phenotypic advantage, supporting the use of 20 nM PMA as a sufficient and appropriate condition for macrophage generation. A distinctive feature of our experimental setting is that PMA was maintained in culture not only during the 72 h differentiation phase, but also throughout the subsequent treatments, without a resting period. This choice was based on previous evidence suggesting that PMA withdrawal may favor partial dedifferentiation of THP-1-derived macrophages toward a monocytic phenotype (29). At the same time, because prolonged PMA exposure may bias macrophages toward an inflammatory baseline state, we specifically asked whether continuous PMA presence could interfere with the acquisition of an IL4-responsive immunoregulatory/tissue-repair program. Our results indicate that this was not the case. Despite continuous PMA exposure, IL4-treated cells retained the ability to upregulate markers associated with this activation state, including IRF4, STAT6, MRC1, and CD274, showed increased CD163 protein expression, and displayed a secretory shift characterized by increased IL10 release together with reduced TNF-α and IL6 secretion. Importantly, macrophages maintained in the M0 condition under the same continuous low-dose PMA exposure did not display the inflammatory enrichment observed after exposure to melanoma SC-CM. Collectively, these findings indicate that continuous low-dose PMA exposure does not abolish macrophage plasticity in our system and supports the use of this protocol as a controlled and suitable platform to investigate melanoma-derived macrophage education. Thus, while not intended to mirror the full physiological spectrum of human TAM biology, this standardized THP-1/PMA system provides a useful experimental setting in which melanoma-derived cues can be interrogated under controlled conditions.

Before addressing macrophage reprogramming, we asked whether melanoma SCs could actively promote monocyte recruitment. This hypothesis was prompted by the marked secretion of CCL2 by both melanosphere models, as indicated by multiplex profiling. Consistent with this, CM from both melanoma SC populations significantly increased THP-1 migration, with a stronger effect observed in response to A375 SC-CM. Importantly, pharmacological inhibition of CCR2 significantly reduced migration induced by both CM, indicating that the CCL2-CCR2 axis contributes, at least in part, to the chemoattractant activity of melanoma SCs. These findings suggest that melanoma SCs may shape the myeloid compartment already at the recruitment stage, by promoting monocyte influx through soluble cues before subsequently influencing macrophage cell-state programs. This, in turn, raises the key question of how these recruited cells are subsequently instructed once exposed to the melanoma SC secretome.

A crucial question explored in this study was the nature of the macrophage phenotype induced by exposure to melanoma SC-CM. TAMs have long been described as predominantly skewed toward an M2-like state within the traditional M1/M2 classification (39). However, this binary model is now widely recognized as an oversimplification, particularly in light of the growing body of single-cell transcriptomic studies showing that intratumoral macrophages comprise highly heterogeneous and dynamically regulated populations. Within this more contemporary view, our data indicate that macrophages exposed to melanoma SC-CM do not adopt a single, easily classifiable state. Rather, based on ssGSEA analysis using literature-derived TAM signatures, they acquire a composite phenotype characterized by the concomitant enrichment of interferon-primed and inflammatory cytokine-enriched programs, together with pro-angiogenic and immune-regulatory features. This pattern was observed in macrophages educated by CMs from both melanoma SC models, suggesting that, despite the biological differences between the two cell lines, SC melanoma states may converge on a partially shared macrophage-education program. The coexistence of inflammatory, angiogenic, and immune-regulatory traits indicates that these macrophages are better understood as occupying a complex tumor-educated state rather than a discrete polarization endpoint. Importantly, a similar overall picture emerged when macrophage phenotypes were assessed using signatures derived from independent single-cell RNA-seq data, further supporting the view that melanoma SC-educated macrophages acquire a multifaceted phenotype. More specifically, this analysis indicated enrichment of an FCN1+IL1B+ inflammatory TAM-like program together with depletion of the FOLR2+SEPP1+ program, suggesting a shift away from resident/homeostatic macrophage features. Consistently, GSEA revealed the simultaneous enrichment of inflammatory signaling pathways together with IL-4- and IL-10-associated programs, further highlighting the complexity of the induced phenotype. Collectively, these findings support the idea that melanoma SCs induce a transcriptionally composite macrophage program in which inflammatory activation coexists with traits linked to immune regulation and tissue remodeling.

Functionally, our data suggest that the macrophage state induced by SC-CM, despite its clearly inflammatory imprint, may not be associated with effective antitumor activity. Indeed, CM from macrophages educated by both melanoma SC models impaired NK-cell cytotoxicity, indicating that the induced macrophage program can exert immunosuppressive effects on innate effector function. In this context, it is important to note that NK-cell cytotoxicity was used as a functional readout of the immunomodulatory activity exerted by melanoma SC-educated macrophages, rather than to define the full spectrum of immune effector populations affected by these cells. This choice was supported by the well-established crosstalk between macrophages and NK cells (40). The loss of this inhibitory activity after heat treatment further suggests that this effect is mediated, at least in part, by heat-labile protein components present in macrophage-CM, probably cytokines and/or chemokines, although the contribution of additional soluble factors cannot be excluded (34, 41).

To gain an initial indication of whether the macrophage program identified *in vitro* might also have *in vivo* relevance, we focused on the transcriptional changes shared by macrophages educated by SC-CM from both melanoma models. The resulting LASSO score, when applied to an independent cohort of primary melanomas, was associated with the same inflammatory macrophage programs identified *in vitro*, supporting the idea that the transcriptional axis induced by melanoma SC-CM may also be represented in human tumors. Although exploratory, the survival analysis further suggested that patients classified as SC-educated macrophage-like showed shorter survival than those classified as M0 macrophage-like. Taken together, these findings suggest that the macrophage program induced *in vitro* by melanoma SC-CM may capture a biologically relevant inflammatory macrophage state that is also detectable *in vivo*. This interpretation is consistent with increasing evidence indicating that inflammatory macrophage programs may support tumor progression rather than counteract it. In melanoma, pro-inflammatory macrophage-derived signals have been shown to induce a protumoral inflammatory state in melanoma cells through TNFR1-IKK-NF-kB signaling, promoting matrix remodeling and invasion (42). Moreover, recent data from primary cutaneous melanoma indicate that TAMs with an inflammatory cytokine-producing phenotype are enriched in metastasizing tumors and are associated with shorter survival (43). More broadly, IL-1β+ TAMs are increasingly recognized as a myeloid state linked to pathogenic inflammation, immune evasion, and resistance to therapy (14). Within this framework, our findings support the view that melanoma SCs induce an inflammatory and tumor-supportive macrophage program, rather than a macrophage state that can be simply interpreted as antitumoral based on its inflammatory features alone.

We next asked whether macrophages previously educated by melanoma SCs feed back on SC-enriched melanoma populations. Under our experimental conditions, macrophage-CMs did not produce evident changes in melanosphere growth or formation but induced selective modulations in the expression of genes previously found to be upregulated in the 3D versus 2D comparison and associated with melanoma stemness-related programs. This response was cell line-dependent, suggesting that the effects of macrophage-derived factors are shaped not only by the phenotype acquired by tumor-educated macrophages, but also by the intrinsic transcriptional background of the melanoma cells themselves. In A375 SCs, the increase in KLF4 and SOX10 may be consistent with reinforcement of a melanoma cell state retaining melanocytic/proliferative features, in line with the established association of SOX10 with melanoma growth and melanocytic identity (44, 45). By contrast, in WM115 SCs, the reduction of KLF4 together with the increase in SOX9 points to a distinct form of transcriptional adaptation rather than to a uniform attenuation of stemness. Indeed, given the context-dependent role of KLF4 in melanoma and the established link of SOX9 with invasive programs and phenotype switching (46, 47), this pattern may reflect a selective rebalancing of melanoma cell-state regulators toward a more adaptive and plastic configuration. Such responses might be interpreted as an adaptation to macrophage-derived microenvironmental cues, consistent with the view that stemness in melanoma is not a fixed property but a dynamic state that can be reshaped while preserving core stemness-associated features. Overall, these findings support the idea that macrophage-derived signals do not uniformly amplify stemness but rather remodel melanoma cell-state regulators in a context-dependent manner according to the intrinsic features of each model.

Although direct evidence linking macrophage-derived inflammatory signals to the regulation of melanoma SCs remains limited, studies in other tumor types support the biological plausibility of such an interaction. In hepatocellular carcinoma, tumor-associated macrophage-derived IL-6 has been shown to promote cancer SCs expansion through STAT3 signaling (48), whereas in basal cell carcinoma macrophage-driven inflammatory signaling has been implicated in tumor cell invasion and angiogenesis (49). In melanoma, an analogous macrophage-to-SC axis has not yet been clearly established. However, inflammatory macrophage-derived cues have been shown to induce protumoral inflammatory and invasive programs in melanoma cells (42, 50, 51). Moreover, IL-6 signaling has been implicated in pro-metastatic pathways involving regulation of WNT-related mediators such as WNT5A (52), supporting the idea that inflammatory myeloid signals can actively reshape tumor cell behavior. Therefore, our data suggest the existence of a reciprocal dialogue in which melanoma SCs educate macrophages and, in turn, receive signals capable of modulating transcriptional programs associated with melanoma cell plasticity. At present, these observations should be considered as preliminary and hypothesis-generating, as they do not demonstrate a net increase in aggressiveness or stemness, but they do support the possibility that melanoma SCs and tumor-educated macrophages participate in a bidirectional signaling circuit.

## 5 Conclusions

In the present study, we show that melanosphere cultures enriched for stemness-associated features actively shape the monocyte-macrophage compartment at multiple levels. Their CMs promoted monocyte recruitment, reprogrammed THP-1-derived macrophages toward a shared but non-canonical inflammatory and immunomodulatory state, remodeled macrophage secretory activity, reduced NK-cell cytotoxicity through heat-labile soluble mediators and fed back on melanoma cell transcriptional programs. Taken together, these findings support a model in which melanoma SC states may contribute to tumor progression not only through cell-intrinsic plasticity, but also by instructing the behavior of myeloid cells in the surrounding environment.

Although obtained in a controlled *in vitro* setting, these results provide a useful framework for dissecting bidirectional interactions between melanoma SCs and macrophages and generate several testable hypotheses. In particular, future studies should determine the extent to which monocyte recruitment and macrophage education depend on the CCL2-CCR2 axis, identify the soluble mediators responsible for the inhibitory effect of SC-educated macrophages on NK-cell cytotoxicity, and clarify whether the transcriptional changes induced in melanoma SCs are accompanied by functional consequences in terms of plasticity, invasion, therapy tolerance, or long-term self-renewal. Extending these analyses to primary monocyte-derived macrophages, primary NK cells, multicellular co-culture systems, and patient-derived melanoma specimens will be important to establish how the macrophage states identified here relate to the complexity of human disease. Overall, our findings provide a biologically grounded basis for further investigating how melanoma cell-state heterogeneity is translated into myeloid remodeling and immune dysfunction.

## Data Availability

The datasets generated during the current study are available in the Gene Expression Omnibus (GEO) repository (https://www.ncbi.nlm.nih.gov/gds) under the accession number GSE329470.

## Supplementary material

Supplementary Table 1 Gene sets utilized for GSEA analysis.

Supplementary Table 2 Differential gene expression and pathway enrichment analyses of melanoma cells grown in 3D versus 2D culture conditions. Lists of differentially expressed genes and significantly enriched pathways identified in the comparison between melanoma cells cultured in 3D and matched 2D conditions.

Supplementary Table 3 Differentially expressed genes and pathway enrichment analyses in macrophages exposed to melanoma SC-conditioned media. Lists of differentially expressed genes and significantly enriched pathways identified in macrophages exposed to melanoma SC-CM compared with M0 macrophages.

Supplementary Table 4 Functional annotation of curated macrophage-state signatures by Metascape. Metascape enrichment analysis of the curated macrophage-state signatures derived from the single-cell RNA-seq dataset (GSE115978), including FCN1+IL1B+ TAMs, C1QC+APOE+ TAMs, and FOLR2+SEPP1+ TAMs. For each signature, the table reports the top enriched functional categories and pathways.

Supplementary Figure 1 Evaluation of the ability of melanoma SC-educated macrophages to modulate melanoma SC phenotype. **(A)** Schematic representation of the experimental procedure to obtain macrophage CM. The image was created by BioRender. **(B)** Representative micrographs of A375 and WM115-derived SCs exposed to CM of M0 or of macrophages previously exposed to A375 and WM115 SC-CM. Magnification 10x.
