# Supplementary material for: Melanoma stem cells drive macrophage reprogramming to a hybrid phenotype, modulating melanoma stemness and compromising NK cell-mediated immunity
Source: Front Immunol. 2026 Jun 2;17:1698412. doi: 10.3389/fimmu.2026.1698412 (PMC13269258; doi:10.3389/fimmu.2026.1698412)
Supplement: Supplementary Table 1 — Gene sets utilized for GSEA analysis. [file Table1.docx]

**Supplementary Table 1. Gene sets utilized for GSEA analysis**

| **Gene set name** | **Genes** | **Ref.** |
| --- | --- | --- |
| **Melanoma stemness** | NGFR, ALDH1A3, ALDH1A1, ABCB5, ABCG2, PROM1, SOX2, KLF4, POU5F1, NANOG, KDM5B, RXRG | (1,2) |
| **Interferon-primed TAMs**  (**IFN**) | CASP1, CASP4, CCL2, CCL3, CCL4, CCL7, CCL8, CD274, CD40, CXCL2, CXCL3, CXCL9, CXCL10, CXCL11, IDO1, IFI6, IFIT1, IFIT2, IFIT3, IFITM1, IFITM3, IRF1, IRF7, ISG15, LAMP3, PDCD1LG2, TNFSF10, C1QA, C1QC, CD38, IL4I1, IFI44L, STAT1 | (3) |
| **Inflammatory cytokine-enriched TAMs**  (**Inflamm**) | CCL2, CCL3, CCL4, CCL5, CCL20, CCL3L1, CCL3L3, CCL4L2, CCL4L4, CXCL1, CXCL2, CXCL3, CXCL5, CXCL8, G0S2, IL1B, IL1RN, IL6, INHBA, KLF2, KLF6, NEDD9, PMAIP1, S100A8, S100A9, SPP1, EGR3, IKZF1, NFKB1, NFE2L2, REL | (3) |
| **Pro-angiogenic TAMs**  (**Angio**) | ADAM8, AREG, BNIP3, CCL2, CCL4, CCL20, CD163, CD300E, CD44, CD55, CEBPB, CLEC5A, CTSB, EREG, FCN1, FLT1, FN1, HES1, IL1B, IL1RN, CXCL8, MAF, MIF, NR1H3, OLR1, PPARG, S100A8, S100A9, S100A12, SERPINB2, SLC2A1, SPIC, SPP1, THBS1, TIMP1, VCAN, VEGFA, BACH1, FOSL2, HIF1A, KLF5, NFKB1, RUNX1, TEAD1, ZEB2 | (3) |
| **Immune regulatory TAMs**  (**Reg**) | CCL2, CD274, CD40, CD80, CD86, CHIT1, CX3CR1, HLA-A, HLA-C, HLA-DQA1, HLA-DQB1, HLA-DRA, HLA-DRB1, HLA-DRB5, ICOSLG, IL10, ITGA4, LGALS9, MARCO, MRC1, TGFB2 | (3) |
| **Lipid-associated TAMs**  (**LA**) | ACP5, APOE, APOC1, ATF1, C1QA, C1QB, C1QC, CCL18, CD163, CD36, CD63, CHI3L1, CTSB, CTSD, CTSL, F13A1, FABP5, FOLR2, GPNMB, IRF3, LGALS3, LIPA, LPL, MARCO, MERTK, MMP7, MMP9, MMP12, MRC1, NR1H3, NRF1, NUPR1, PLA2G7, RNASE1, SPARC, SPP1, TFDP2, TREM2, ZEB1, FOS, JUN, HIF1A, MAF, MAFB, TCF4, TFEC | (3) |
| **Proliferating TAMs**  (**Prolif**) | CCNA2, CDC45, CDK1, H2AC13, H4C3, HMGB1, HMGN2, MKI67, RRM2, STMN1, TOP2A, TUBA1B, TUBB, TYMS | (3) |
| **FCN1+IL1B+ TAMs** | CD300E, IL1B, VCAN, FCN1, S100A8, TREM1, APOBEC3A, ANPEP, FGR, SLC11A1, BCL2A1, ALOX5, LYZ | (4) |
| **FOLR2+SEPP1+ TAMs** | SELENOP, HPGDS, FOLR2, SLC40A1, SPP1, LGMN, GPR34, DAB2, F13A1, NRP1, STAB1, VSIG4, MS4A4A, FCGRT, ALDH1A1, PLTP, MAF, CD209, SLCO2B1, GRN | (4) |
| **C1QC+APOE+ TAMs** | NR1H3, C1QA, C1QB, C1QC, APOE, APOC1, SERPING1, C2, GPNMB, IL4I1, LILRB4, SLAMF8, FCGR1A, HLA-DRA, HLA-DPA1, CD74, TMEM176A, TMEM176B | (4) |

**References**

1. Marzagalli M, Raimondi M, Fontana F, Montagnani Marelli M, Moretti RM, et al. Cellular and molecular biology of cancer stem cells in melanoma: Possible therapeutic implications. *Semin Cancer Biol* (2019) Dec;59:221-235. doi: 10.1016/j.semcancer.2019.06.019.

2. Parmiani G. Melanoma Cancer Stem Cells: Markers and Functions. *Cancers (Basel)* (2016) Mar 11;8(3):34. doi: 10.3390/cancers8030034.

3. Ma RY, Black A, Qian BZ. Macrophage diversity in cancer revisited in the era of single-cell omics. *Trends Immunol* (2022) Jul;43(7):546-563. doi: 10.1016/j.it.2022.04.008.

4. Jerby-Arnon L, Shah P, Cuoco MS, Rodman C, Su MJ, Melms JC, et al. A Cancer Cell Program Promotes T Cell Exclusion and Resistance to Checkpoint Blockade. *Cell* (2018) Nov 1;175(4):984-997.e24. doi: 10.1016/j.cell.2018.09.006.
