# Supplementary figures and images for: Melanoma stem cells drive macrophage reprogramming to a hybrid phenotype, modulating melanoma stemness and compromising NK cell-mediated immunity
Source: Front Immunol. 2026 Jun 2;17:1698412. doi: 10.3389/fimmu.2026.1698412 (PMC13269258; doi:10.3389/fimmu.2026.1698412)

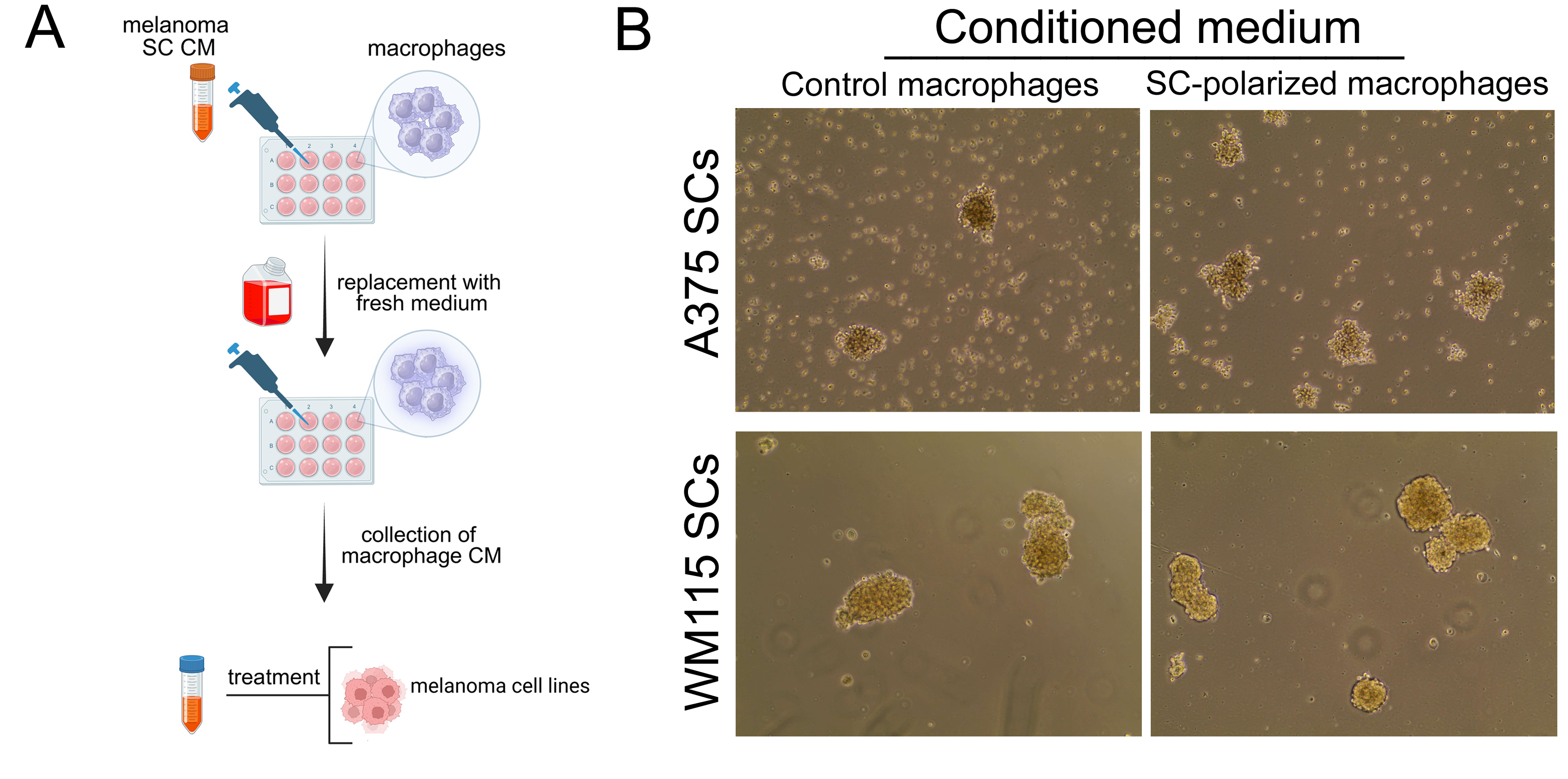

Supplement: Supplementary Figure 1 — Evaluation of the ability of melanoma SC-educated macrophages to modulate melanoma SC phenotype. (A) Schematic representation of the experimental procedure to obtain macrophage CM. The image was created by BioRender. (B) Representative micrographs of A375 and WM115-derived SCs exposed to CM of M0 or of macrophages previously exposed to A375 and WM115 SC-CM. Magnification 10x. [file Image1.tif]
